# Nonlinear electromechanical analysis of axisymmetric thin circular plate based on flexoelectric theory

**DOI:** 10.1038/s41598-021-01289-0

**Published:** 2021-11-05

**Authors:** Xue Ji

**Affiliations:** Department of Industrial Engineering, School of Management Engineering, Shandong Jianzhu University, Jinan, 250101 People’s Republic of China

**Keywords:** Engineering, Materials science, Nanoscience and technology, Physics

## Abstract

Flexoelectricity will dominate the electromechanical coupling of intelligent components in MEMS/NEMS due to its size-dependency. This paper focuses on investigating the flexoelectric responses of intelligent components of the circular plate type, which are commonly used in MEMS/NEMS. Utilizing Hamilton’s principle, the nonlinear flexoelectric circular plate model is presented by combining von Kármán plate theory and flexoelectric theory. The equilibrium equations and all boundary conditions are obtained and then discretized. The nonlinear static bending of the simply supported axisymmetric flexoelectric circular plate is investigated by combining DQM and iteration method. The distributions of dimensionless bending deflection and electric potential are analyzed under different loads. Moreover, the nonlinear free vibration behaviors are also investigated by combining the Galerkin method and Lindstedt–Poincaré Method. The flexoelectric effect and stiffening effect of strain gradient are revealed. This paper will be helpful to promote the application of flexoelectric intelligent components of the circular plate type, which are encountered commonly in engineering.

## Introduction

Intelligent components play a leading role in Micro-/Nano-Electro-Mechanical System (MEMS/NEMS). In general, intelligent components rely on electromechanical coupling effects to perform specific functions, such as piezoelectric effect. However, recently, flexoelectric effect has received a lot of attention. Flexoelectric effect is a kind of higher-order electromechanical coupling effect, which has a great prospect in the field of sensing, actuating, energy harvesting and so on^1,2^. The advantage of flexoelectric effect is that, on the one hand, it exists in all dielectric materials, which broadens the choice of suitable materials for electromechanical applications, and, on the other hand, it is size-dependent which will be enhanced in the nano scale.

Flexoelectric effect includes its direct effect and inverse effect^3^. A polarization can be induced by strain gradient in the direct flexoelectric effect, and elastic stresses can also be induced by polarization gradient in the inverse flexoelectric effect. Many experiments have investigated the flexoelectric effect in ceramics^4–6^, polymers^7^, biological materials^8^ and semiconductor^9^. Shu et al. summarized the measurement methods of flexoelectric coefficients^1^. A non-polarized PVDF curved flexoelectric actuator with high displacement resolution has been designed by Zhang et al.^10^. Kwon et al.^11^ fabricated a flexoelectric microphone possessing a wide working frequency range and a high sensitivity simultaneously. By fabricating a silicon-compatible flexoelectric cantilever actuator, Bhaskar et al.^12^ demonstrated that flexoelectricity is an alternative to lead-free MEMS and NEMS.

For capturing the flexoelectric effect, the continuum theory for flexoelectricity in dielectric has been proposed on the basis of thermodynamics. Tagantsev developed the flexoelectric theory of solid crystalline dielectrics^13^. Sahin and Dost choose the deformation gradient, the first and second gradients of deformation gradient, the polarization and polarization gradient as independent variables to describe both the direct and converse flexoelectric couplings^14^. However, the plethora of parameters restricted its applications. Hence, by taking the strain, polarization and their gradients as independent variables, Maranganti et al.^15^ proposed a modified flexoelectric theory. The variational principle of flexoelectric theory has been developed by Hu and Shen^16^ and the corresponding governing equations for flexoelectricity were derived. Then the surface effects were introduced and the electric enthalpy variational principle with surface effects was presented^17^. Furthermore, the flexoelectric theory has been reformulated by Li et al.^18^ by introducing the strain gradient orthogonal components. In addition, the flexoelectric theories considering rotation gradient effect rather than strain gradient effect have also been presented^19,20^. Deng et al. developed a nonlinear theoretical framework for flexoelectricity in soft materials^21^. The generalized form of the bias-strain-concentration equation has been derived by Morozovska et al. for mixed ionic-electronic conductors^22^. Moreover, Wang et al.^3^ reviewed the development of flexoelectric theories in solids and an overview of numerical procedures on modeling flexoelectricity has been provided by Zhuang et al.^23^.

According to the flexoelectric theory, Li et al. analyzed the flexoelectric effects of an isotropic bar in compression, torsion and bending^24^. Qi et al. presented a curved flexoelectric beam model and analyzed the flexoelectric responses^25^. Gabr and El Dhaba extended the isotropic beams to anisotropic beams with cubic symmetry and investigated the flexoelectric response in bending^26^. By introducing surface and thermal effects, Barati has studied the nonlinear behavior of flexoelectric beam in vibration^27^. The dynamic flexoelectric actuation of a cantilever actuated by the inverse flexoelectric effect has been evaluated by Fan et al.^28^. Bagheri and Beni studied the forced vibration of viscoelastic/flexoelectric nanobeams^29^. Baroudi and Najar revealed the effects of geometric nonlinearity on the static and dynamic responses of piezoelectric flexoelectric nanobeams^30^. Dai et al. analyzed the output performance of flexoelectric beam energy harvester with the consideration of geometric nonlinearity^31^. The size-dependent dynamic pull-in instability of micro/nanobeams with considering von Kármán hypothesis has been investigated according to the nonlocal strain gradient theory tuned by flexoelectric and piezoelectric effects^32^.

In addition, the flexoelectric responses of circular plate have been investigated by Li et al.^33^. Amir et al. presented a dynamic model of nanocomposite sandwich plates resting on Pasternak foundation^34^. The nonlinear functionally graded sandwich porous core nano-plates model has been proposed by Zeng et al.^35^. The nonlinear behavior of functionally graded flexoelectric nano-plate has been studied by Ghobadi et al. in thermal environment^36^. Moreover, the energy harvesting performance of a flexoelectric plate attached to an elastic substrate was also investigated and the optimal inner and outer radii were identified for maximum power output^37^.

As the representative intelligent components in MEMS/NEMS, flexoelectric circular plate lacks enough attention. As far as we know, the flexoelectricity in nonlinear behaviors of circular plate is still absent. This paper is devoted to investigating the electromechanical coupling behavior of intelligent components of the circular plate type in large deflection. On the basis of the isotropic flexoelectric theory reviewed in the next Section, the flexoelectric model of circular plate in large deflection deformation is presented in “Nonlinear flexoelectric model for circular plate”. Then by applying the differential quadrature method (DQM) to discretize the current model in “Nonlinear bending of flexoelectric circular plate”, the nonlinear static bending properties of a flexoelectric plate in simply supported boundary conditions are investigated. “Nonlinear free vibration of axisymmetric flexoelectric circular plate” studied the nonlinear free vibration properties of a flexoelectric plate by combining the Galerkin method and Lindstedt–Poincaré Method. Finally, “Conclusion” obtained the conclusion. It is hoped that this paper will be helpful to promote the application of flexoelectric intelligent components of the circular plate type.

## Review of the isotropic flexoelectric theory

In flexoelectric theory, the internal energy density *U* can be expressed as a function of independent variables which includes strain gradient *η*_*ijk*_ and polarization gradient *Q*_*ij*_ except to the traditional strain *ε*_*ij*_ and polarization *P*_*i*_, written as *U* = *U*(*ε*_*ij*_, *P*_*i*_, *η*_*ijk*_, *Q*_*ij*_)^18^. The strain *ε*_*ij*_, the strain gradient *η*_*ijk*_ and the polarization gradient *Q*_*ij*_ are defined, respectively, as

1 $$\varepsilon_{ij} = \frac{1}{2}(u_{i,j} + u_{j,i} ),\,\,\eta_{ijk} = \varepsilon_{jk,i} ,\,\,Q_{ij} = P_{i,j} ,$$

where a comma means differentiation with respect to coordinates and *u*_*i*_ represents displacement component. For isotropic dielectrics, the general expression of flexoelectric internal density involves five higher-order material constants associated with the coupling of strain gradient to strain gradient^24^. However, the plethora of higher-order material constants restricts the application of this theory. For simplicity, the flexoelectric theory with rotation gradient effect has also been proposed^19,20^. Although the number of higher-order material constants has decreased, the effect of other strain gradient components has also been neglected due to the fact that rotation gradient is only the anti-symmetric part of strain gradient. Obviously, it is essential to consider all strain gradient effect, but the number of higher-order material constants need to be decreased for applications.

On the basis of general flexoelectric theory including all strain gradient effects, Li et al.^18^ reformulated its internal energy density by introducing two sets of independent strain gradient components and demonstrated that there are three independent higher-order material constants associated with the coupling of strain gradient to strain gradient. The same conclusion has also been obtained by Zhou et al.^38^ in the strain gradient elasticity theory. Then, Ji et al.^39^ summarized and revisited the decomposition scheme of strain gradient tensor and found that, as a matter of fact, the strain gradient consists of three strain gradient components, $\eta_{ijk} = \eta_{ijk}^{V} + \eta_{ijk}^{(1)} + \eta_{ijk}^{as}$, in which the strain gradient spherical component $\eta_{ijk}^{V}$, the deviatoric stretch gradient component $\eta_{ijk}^{(1)}$ and the symmetric rotation strain gradient component $\eta_{ijk}^{as}$ are written as

2 $$\begin{aligned} \eta_{ijk}^{V} & = \frac{1}{10}\delta_{ij} (3\varepsilon_{km,m} - \varepsilon_{mm,k} ) + \frac{1}{10}\delta_{ki} (3\varepsilon_{jm,m} - \varepsilon_{mm,j} ) + \frac{1}{5}\delta_{jk} (2\varepsilon_{mm,i} - \varepsilon_{im,m} ) \\ \eta_{ijk}^{(1)} & = \frac{1}{3}(\varepsilon_{jk,i} + \varepsilon_{ik,j} + \varepsilon_{ij,k} ) \\ & \quad - \frac{1}{15}[\delta_{ij} (2\varepsilon_{km,m} + \varepsilon_{mm,k} ) + \delta_{ki} (2\varepsilon_{jm,m} + \varepsilon_{mm,j} ) + \delta_{kj} (2\varepsilon_{im,m} + \varepsilon_{mm,i} )] \\ \eta_{ijk}^{as} & = \frac{1}{6}[2\varepsilon_{jk,i} - \varepsilon_{ik,j} - \varepsilon_{ij,k} + (e_{ijp} e_{kmn} + e_{ikp} e_{jmn} )\varepsilon_{np,m} ] \\ \end{aligned}$$

with *e*_*ijk*_ and *δ*_*ij*_ denoting the alternating tensor and Kronecker delta, respectively. According to the three strain gradient components, the internal energy density of Li et al. is re-written as

3 $$\begin{aligned} U & = \frac{1}{2}k\varepsilon_{ii} \varepsilon_{jj} + \mu \varepsilon^{\prime}_{ij} \varepsilon^{\prime}_{ij} + 3\mu l_{0}^{2} \eta_{ijk}^{V} \eta_{ijk}^{V} + \mu l_{1}^{2} \eta_{ijk}^{(1)} \eta_{ijk}^{(1)} + 3\mu l_{2}^{2} \eta_{ijk}^{as} \eta_{ijk}^{as} \\ & \quad + \frac{1}{2}\alpha (P_{i} P_{i} + \beta_{1}^{2} Q_{nn} Q_{mm} + \beta_{2}^{2} Q_{ij} Q_{ij} + \beta_{3}^{2} Q_{ij} Q_{ji} ) \\ & \quad + f_{1} P_{i} \eta_{ijj}^{V} + 2f_{2} P_{i} \eta_{jji}^{V} - f_{1} \varepsilon_{nn} Q_{mm} - 2f_{2} \varepsilon_{ij} Q_{ij} \\ \end{aligned}$$

in which $\varepsilon^{\prime}_{ij} = \varepsilon_{ij} - \tfrac{1}{3}\delta_{ij} \varepsilon_{nn}$ is the deviatoric strain tensor, *k* and *μ* denote the bulk and shear modulus, *α* denotes the reciprocal dielectric susceptibility, *l*_*i*_(*i* = 0,1,2) and *β*_*i*_(*i* = 1,2,3) represent the length-scale parameters related to strain gradient and polarization gradient, respectively, *f*_1_ and *f*_2_ represent the flexocoupling coefficients. It is found that only the spherical component $\eta_{ijk}^{V}$ can induce polarization in isotropic dielectrics^40^.

Accordingly, the stress *σ*_*ij*_, the higher-order stresses $\tau_{ijk}^{V}$, $\tau_{ijk}^{(1)}$,$\tau_{ijk}^{as}$, the effective local electric field *E*_*i*_ and higher-order electric field *V*_*ij*_ can be given by the constitutive equations,

4 $$\sigma_{ij} = k\delta_{ij} \varepsilon_{nn} + 2\mu \varepsilon^{\prime}_{ij} - f_{1} \delta_{ij} Q_{kk} - f_{2} (Q_{ij} { + }Q_{ji} ),$$

5 $$\tau_{ijk}^{V} = 6\mu l_{0}^{2} \eta_{ijk}^{V} + f_{1} \delta_{jk} P_{i} + f_{2} (\delta_{ij} P_{k} { + }\delta_{ik} P_{j} ),\tau_{ijk}^{(1)} = 2\mu l_{1}^{2} \eta_{ijk}^{(1)} ,\tau_{ijk}^{as} = 6\mu l_{2}^{2} \eta_{ijk}^{as} ,$$

6 $$E_{i} = \alpha P_{i} + f_{1} \eta_{ijj}^{V} + 2f_{2} \eta_{jji}^{V} ,$$

7 $$V_{ij} = \alpha (\delta_{ij} \beta_{1}^{2} Q_{kk} + \beta_{2}^{2} Q_{ij} + \beta_{3}^{2} Q_{ji} ) - f_{1} \delta_{ij} \varepsilon_{nn} - 2f_{2} \varepsilon_{ij} .$$

Based on Eqs. (4)–(7), the internal energy density Eq. (3) can also be expressed as

8 $$U = \frac{1}{2}\sigma_{ij} \varepsilon_{ij} + \frac{1}{2}\tau_{ijk}^{V} \eta_{ijk}^{V} + \frac{1}{2}\tau_{ijk}^{(1)} \eta_{ijk}^{(1)} + \frac{1}{2}\tau_{ijk}^{as} \eta_{ijk}^{as} + \frac{1}{2}E_{i} P_{i} + \frac{1}{2}V_{ij} Q_{ij} .$$

The present flexoelectric theory considers all strain gradient effects, polarization gradient effects, direct and inverse flexoelectric effects, and only three material length scale parameters associated with strain gradient are included, which is a general theory with practicability. When the electrical terms are neglected, the flexoelectric theory will reduce to the general strain gradient theory^41^.

## Nonlinear flexoelectric model for circular plate

**Figure 1:**
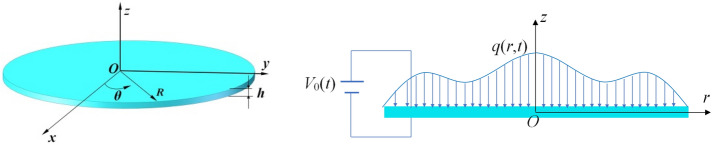
Schematic of axisymmetric flexoelectric circular plate.

Consider a flexoelectric circular plate as shown in Fig. 1. The thickness of plate is *h* and the radius is *R.* The current circular plate is subjected to a transverse load *q* and an electric voltage *V*_0_ between its upper and lower surfaces. The circular plate with specific boundary conditions deforms under the action of external load. A cylindrical coordinate system is adopted to establish its static and dynamic models where *rθ* plane is consistent with the plate midplane. According to the Kirchhoff assumption, the *r*-, *θ*-, *z*-displacement components are given by

9 $$u_{r} (r,z,t) = u(r,t) - z\frac{\partial w}{{\partial r}},u_{\theta } (r,z) = 0,u_{z} (r,z) = w(r,t),$$

where *w* denotes the transverse deflection, *u* represents the radial displacement in midplane and *t* is time. Moreover, the polarization is assumed to be along the thickness direction, given by

10 $$P_{z} = P_{z} (r,z,t).$$

From the Von Kármán’s strain theory, the non-vanishing strains take the form

11 $$\varepsilon_{rr} = \frac{\partial u}{{\partial r}} + \frac{1}{2}\left( {\frac{\partial w}{{\partial r}}} \right)^{2} - z\frac{{\partial^{2} w}}{{\partial r^{2} }},\varepsilon_{\theta \theta } = \frac{u}{r} - \frac{z}{r}\frac{\partial w}{{\partial r}}.$$

Substituting the nonzero strains Eq. (11) into Eq. (2), these three orthogonal strain gradient components $\eta_{ijk}^{V}$, $\eta_{ijk}^{(1)}$ and $\eta_{ijk}^{as}$ can be obtained and shown in Appendix A. Furthermore, from constitutive relations Eqs. (6) and (7), it can be obtained that,

12 $$E_{z} = \alpha P_{z} - f_{1} \left( {\frac{{\partial^{2} w}}{{\partial r^{2} }} + \frac{\partial w}{{r\partial r}}} \right),$$

13 $$V_{zz} = \alpha (\beta_{1}^{2} + \beta_{2}^{2} + \beta_{3}^{2} )Q_{zz} - f_{1} \left( {\frac{\partial u}{{\partial r}} + \frac{u}{r} + \frac{1}{2}\left( {\frac{\partial w}{{\partial r}}} \right)^{2} - z\frac{{\partial^{2} w}}{{\partial r^{2} }} - z\frac{\partial w}{{r\partial r}}} \right).$$

According to the general governing equations for electroelastic analysis of dielectrics^18^, *E*_*z*_ and *V*_*zz*_ in Eqs. (12) and (13) satisfy

14 $$\begin{aligned} & E_{z} - V_{zz,z} + \varphi_{,z} = 0 \\ & - \varepsilon_{0} \varphi_{,zz} + P_{z,z} = 0 \\ \end{aligned}$$

in which *φ* is the potential of the Maxwell self-field. It should be noted here that the higher-order electric field *V*_*zr*_ is ignored in comparison with *V*_*zz*_ due to the radial gradient is much smaller than the transverse gradient for thin circular plate. Substituting Eqs. (12) and (13) into Eq. (14), the equilibrium equations for electroelastic analysis of dielectrics are rewritten as

15 $$\left\{ \begin{gathered} \varphi_{,z} = \alpha \beta^{2} \frac{{\partial^{2} P_{z} }}{{\partial z^{2} }} - \alpha P_{z} + 2f_{1} \left( {\frac{{\partial^{2} w}}{{\partial r^{2} }} + \frac{\partial w}{{r\partial r}}} \right) \hfill \\ - \varepsilon_{0} \varphi_{,zz} + \frac{{\partial P_{z} }}{\partial z}{ = }0, \hfill \\ \end{gathered} \right.$$

where the parameter $\beta^{2} = \beta_{1}^{2} + \beta_{2}^{2} + \beta_{3}^{2}$ is defined. Combining this governing equations and the electric boundary conditions,

16 $$\begin{aligned} & at\,\, \, z = \frac{h}{2}:V_{zz} = 0,\varphi \left( \frac{h}{2} \right) = V_{0} \\ & at \, \,\,z = - \frac{h}{2}:V_{zz} = 0,\varphi \left( { - \frac{h}{2}} \right) = 0, \\ \end{aligned}$$

the polarization and electric potential are obtained and written as

17 $$\begin{aligned} P_{z} & = \frac{{f_{1} }}{{\alpha \beta^{2} \lambda }}\left( {\frac{\partial u}{{\partial r}} + \frac{u}{r} + \frac{1}{2}\left( {\frac{\partial w}{{\partial r}}} \right)^{2} } \right)\frac{{e^{\lambda z} - e^{ - \lambda z} }}{{e^{\lambda h/2} + e^{ - \lambda h/2} }} \\ & \quad - \frac{{f_{1} h}}{{2\alpha \beta^{2} \lambda }}\left( {\frac{{\partial^{2} w}}{{\partial r^{2} }} + \frac{\partial w}{{r\partial r}}} \right)\frac{{e^{\lambda z} + e^{ - \lambda z} }}{{e^{\lambda h/2} - e^{ - \lambda h/2} }} + \frac{{1 + 2\alpha \varepsilon_{0} }}{{1 + \alpha \varepsilon_{0} }}\frac{{f_{1} }}{\alpha }\left( {\frac{{\partial^{2} w}}{{\partial r^{2} }} + \frac{\partial w}{{r\partial r}}} \right) - \frac{{V_{0} }}{\alpha h}, \\ \end{aligned}$$

18 $$\begin{aligned} \varphi & = \frac{{f_{1} }}{{1 + \alpha \varepsilon_{0} }}\left[ {\frac{\partial u}{{\partial r}} + \frac{u}{r} + \frac{1}{2}\left( {\frac{\partial w}{{\partial r}}} \right)^{2} } \right]\left( {\frac{{e^{\lambda z} + e^{ - \lambda z} }}{{e^{\lambda h/2} + e^{ - \lambda h/2} }} - 1} \right) \\ & \quad + \frac{{f_{1} h}}{{2(1 + \alpha \varepsilon_{0} )}}\left( {\frac{{\partial^{2} w}}{{\partial r^{2} }} + \frac{\partial w}{{r\partial r}}} \right)\left( {\frac{2}{h}z - \frac{{e^{\lambda z} - e^{ - \lambda z} }}{{e^{\lambda h/2} - e^{ - \lambda h/2} }}} \right) + \frac{{V_{0} }}{h}z + \frac{{V_{0} }}{2} \\ \end{aligned}$$

with the parameter *λ* defined as

19 $$\lambda { = }\sqrt {\frac{{1 + \alpha \varepsilon_{0} }}{{\varepsilon_{0} \alpha \beta^{2} }}} .$$

Subsequently, the equilibrium equations and mechanical boundary conditions of the current nonlinear flexoelectric circular plate can be obtained by applying Hamilton’s principle,

20 $$\delta \int_{{t_{1} }}^{{t_{2} }} {\left( { - 2\pi \int_{0}^{R} {\int_{{ - {h \mathord{\left/ {\vphantom {h 2}} \right. \kern-\nulldelimiterspace} 2}}}^{{{h \mathord{\left/ {\vphantom {h 2}} \right. \kern-\nulldelimiterspace} 2}}} {Hr{\text{d}}r{\text{d}}z} } + T + W} \right){\text{d}}t} = 0.$$

The electric enthalpy density *H* is written as

21 $$H = U - \frac{1}{2}\varepsilon_{0} \varphi_{,z} \varphi_{,z} + \varphi_{,z} P_{z}$$

with *ε*_0_ denoting the permittivity of a vacuum and *U* being the internal energy density defined by Eq. (3). The kinetic energy *T* of the axisymmetric circular plate is

22 $$T = \pi \rho h\int_{0}^{R} {(\dot{u}^{2} + \dot{w}^{2} )r{\text{d}}r} ,$$

where *ρ* represents the mass density, $\dot{u}$ and $\dot{w}$ denote the first derivative of radial displacement and deflection versus time, respectively. The work *W* done by the transverse load *q* is

23 $$W = 2\pi \int_{0}^{R} {qw} r{\text{d}}r.$$

Applying the variational principle of Eq. (20), the equilibrium equations of the nonlinear flexoelectric circular plate are derived as

24 $$\begin{aligned} & 2(c_{4} - d_{1} )\left( {\frac{u}{{r^{2} }} - \frac{\partial u}{{r\partial r}} - \frac{{\partial^{2} u}}{{\partial r^{2} }} - \frac{\partial w}{{\partial r}}\frac{{\partial^{2} w}}{{\partial r^{2} }}} \right) - \frac{{c_{4} }}{r}\left( {\frac{\partial w}{{\partial r}}} \right)^{2} + c_{5} \frac{1}{r}\left( {\frac{\partial w}{{\partial r}}} \right)^{2} + 2c_{7} \left[ {\frac{{\partial^{4} u}}{{\partial r^{4} }} + 2\frac{{\partial^{3} u}}{{r\partial r^{3} }}} \right. \\ & \quad \left. { - 3\frac{{\partial^{2} u}}{{r^{2} \partial r^{2} }} + 3\frac{\partial u}{{r^{3} \partial r}} - 3\frac{u}{{r^{4} }} + \frac{{\partial^{2} w}}{{\partial r^{2} }}\left( {2\frac{{\partial^{2} w}}{{r\partial r^{2} }} + 3\frac{{\partial^{3} w}}{{\partial r^{3} }}} \right) + \frac{\partial w}{{\partial r}}\left( {\frac{{\partial^{4} w}}{{\partial r^{4} }} + 2\frac{{\partial^{3} w}}{{r\partial r^{3} }} - 2\frac{{\partial^{2} w}}{{r^{2} \partial r^{2} }}} \right)} \right] \\ & \quad + c_{9} \left( {\frac{\partial w}{{r^{2} \partial r}}\frac{{\partial^{2} w}}{{\partial r^{2} }} - \frac{1}{r}\left( {\frac{{\partial^{2} w}}{{\partial r^{2} }}} \right)^{2} - \frac{1}{r}\frac{\partial w}{{\partial r}}\frac{{\partial^{3} w}}{{\partial r^{3} }}} \right) + \rho \ddot{u} = 0, \\ \end{aligned}$$

25 $$\begin{aligned} & \frac{{c_{7} h^{3} }}{6}\nabla^{6} w - \left( {\frac{{c_{4} h^{3} }}{6} + 2c_{1} h - d_{2} } \right)\nabla^{4} w - 4c_{2} h\left( {\frac{\partial w}{{r\partial r}}} \right)^{2} \left( {\frac{\partial w}{{r\partial r}} - 3\frac{{\partial^{2} w}}{{\partial r^{2} }}} \right) \\ & \quad + (c_{4} - d_{1} )h\left[ {2\frac{{\partial^{2} u}}{{\partial r^{2} }}\frac{\partial w}{{\partial r}} + 2\frac{\partial u}{{\partial r}}\left( {\frac{{\partial^{2} w}}{{\partial r^{2} }} + \frac{\partial w}{{r\partial r}}} \right) + \left( {\frac{\partial w}{{\partial r}}} \right)^{2} \left( {3\frac{{\partial^{2} w}}{{\partial r^{2} }} + \frac{\partial w}{{r\partial r}}} \right)} \right] + 2h(c_{5} - d_{1} )\left( {\frac{\partial u}{{\partial r}}\frac{\partial w}{{r\partial r}} + \frac{u}{r}\frac{{\partial^{2} w}}{{\partial r^{2} }}} \right) \\ & \quad - {2}c_{7} h\left[ {\frac{{\partial^{4} u}}{{\partial r^{4} }}\frac{\partial w}{{\partial r}} + \frac{{\partial^{3} u}}{{\partial r^{3} }}\left( {\frac{{\partial^{2} w}}{{\partial r^{2} }} + 2\frac{\partial w}{{r\partial r}}} \right) + \frac{{\partial^{2} u}}{{\partial r^{2} }}\frac{{\partial^{2} w}}{{r\partial r^{2} }} + 3\frac{\partial w}{{r\partial r}}\left( {\frac{{\partial^{2} w}}{{\partial r^{2} }}} \right)^{2} + 2\left( {\frac{\partial w}{{\partial r}}} \right)^{2} \frac{{\partial^{3} w}}{{r\partial r^{3} }}} \right. \\ & \quad \left. { + \left( {\frac{\partial w}{{\partial r}}} \right)^{2} \frac{{\partial^{4} w}}{{\partial r^{4} }} + \left( {\frac{{\partial^{2} w}}{{\partial r^{2} }}} \right)^{3} + 4\frac{\partial w}{{\partial r}}\frac{{\partial^{2} w}}{{\partial r^{2} }}\frac{{\partial^{3} w}}{{\partial r^{3} }}} \right] + 4c_{8} h\left[ {\frac{{\partial^{2} u}}{{r^{2} \partial r^{2} }}\frac{\partial w}{{\partial r}} + \left( {\frac{\partial u}{{r^{2} \partial r}} - \frac{u}{{r^{3} }}} \right)\left( {\frac{{\partial^{2} w}}{{\partial r^{2} }} - 2\frac{\partial w}{{r\partial r}}} \right)} \right] \\ & \quad + c_{9} h\left[ { - \frac{{\partial^{3} u}}{{\partial r^{3} }}\frac{\partial w}{{r\partial r}} - \frac{{\partial^{2} u}}{{r\partial r^{2} }}\left( {\frac{{\partial^{2} w}}{{\partial r^{2} }} - 3\frac{\partial w}{{r\partial r}}} \right) + 3\left( {\frac{\partial u}{{r^{2} \partial r}} - \frac{u}{{r^{3} }}} \right)\left( {\frac{{\partial^{2} w}}{{\partial r^{2} }} - 2\frac{\partial w}{{r\partial r}}} \right)} \right] - \rho h\ddot{w} + q = 0 \\ \end{aligned}$$

with

26 $$\nabla^{6} = \frac{{\partial^{6} }}{{\partial r^{6} }} + \frac{3}{r}\frac{{\partial^{5} }}{{\partial r^{5} }} - \frac{3}{{r^{2} }}\frac{{\partial^{4} }}{{\partial r^{4} }} + \frac{6}{{r^{3} }}\frac{{\partial^{3} }}{{\partial r^{3} }} - \frac{9}{{r^{4} }}\frac{{\partial^{2} }}{{\partial r^{2} }} + \frac{9}{{r^{5} }}\frac{\partial }{\partial r}\quad \nabla^{4} = \frac{{\partial^{4} }}{{\partial r^{4} }} + \frac{2}{r}\frac{{\partial^{3} }}{{\partial r^{3} }} - \frac{1}{{r^{2} }}\frac{{\partial^{2} }}{{\partial r^{2} }} + \frac{1}{{r^{3} }}\frac{\partial }{\partial r}$$

and the coefficients $c_{i} (i = 1,2,3, \ldots 9)$, *d*_1_ and *d*_2_ are given by

27 $$\begin{aligned} & d_{1} = \frac{{f_{1}^{2} \varepsilon_{0} \lambda }}{{h(1 + \alpha \varepsilon_{0} )}}\frac{{e^{\lambda h/2} - e^{ - \lambda h/2} }}{{e^{\lambda h/2} + e^{ - \lambda h/2} }},d_{2} = \frac{{f_{1}^{2} h}}{{\alpha (1 + \alpha \varepsilon_{0} )}}\left( {1 + \frac{{\lambda h\alpha \varepsilon_{0} }}{2}\frac{{e^{\lambda h/2} + e^{ - \lambda h/2} }}{{e^{\lambda h/2} - e^{ - \lambda h/2} }}} \right) \\ & c_{1} = \frac{6}{5}\mu l_{0}^{2} + \frac{4}{15}\mu l_{1}^{2} + \mu l_{2}^{2} ,c_{2} = \frac{{9}}{{{20}}}\mu l_{0}^{2} + \frac{4}{15}\mu l_{1}^{2} + \frac{1}{4}\mu l_{2}^{2} \\ & c_{3} = \frac{12}{5}\mu l_{0}^{2} - \frac{2}{15}\mu l_{1}^{2} - 2\mu l_{2}^{2} ,c_{4} = \frac{1}{2}\frac{E}{{1 - v^{2} }},c_{5} = \frac{1}{2}\frac{Ev}{{1 - \nu^{2} }} \\ & c_{6} = \frac{6}{5}\mu l_{0}^{2} - \frac{7}{5}\mu l_{1}^{2} ,c_{7} = \frac{9}{5}\mu l_{0}^{2} + \frac{2}{5}\mu l_{1}^{2} ,c_{8} = \frac{3}{5}\mu l_{0}^{2} + \frac{4}{5}\mu l_{1}^{2} ,c_{9} = \frac{6}{5}\mu l_{0}^{2} - \frac{2}{5}\mu l_{1}^{2} \\ \end{aligned}$$

in which *E* and *v* denote the Young’s modulus and the Poisson ratio, respectively. The initial conditions are written as

28 $$\rho h\dot{u}\delta u|_{{t_{1} }}^{{t_{2} }} = 0,\rho h\dot{w}\delta w|_{{t_{1} }}^{{t_{2} }} = 0.$$

The mechanical boundary conditions satisfy

29 $$\begin{aligned} & S_{u} (R)\delta u(R) - S_{u} (0)\delta u(0) = 0,S_{u1} (R)\delta u_{,r} (R) - S_{u1} (0)\delta u_{,r} (0) = 0 \\ & S_{w} (R)\delta w(R) - S_{w} (0)\delta w(0) = 0,S_{w1} (R)\delta w_{,r} (R) - S_{w1} (0)\delta w_{,r} (0) = 0 \\ & S_{w2} (R)\delta w_{,rr} (R) - S_{w2} (0)\delta w_{,rr} (0) = 0, \\ \end{aligned}$$

where *S*_*u*_, *S*_*u*1_, *S*_*w*_, *S*_*w*1_, *S*_*w*2_ are shown in Appendix B.

When we ignore all the nonlinear terms, the nonlinear flexoelectric model will reduce to the corresponding linear model of circular plate. In the linear model, the polarization and electrical potential are shown, respectively, as

30 $$P_{z} = - \frac{{f_{1} h}}{{2\alpha \beta^{2} \lambda }}\left( {\frac{{\partial^{2} w}}{{\partial r^{2} }} + \frac{\partial w}{{r\partial r}}} \right)\frac{{e^{\lambda z} + e^{ - \lambda z} }}{{e^{\lambda h/2} - e^{ - \lambda h/2} }} + \frac{{1 + 2\alpha \varepsilon_{0} }}{{1 + \alpha \varepsilon_{0} }}\frac{{f_{1} }}{\alpha }\left( {\frac{{\partial^{2} w}}{{\partial r^{2} }} + \frac{\partial w}{{r\partial r}}} \right) - \frac{{V_{0} }}{\alpha h},$$

31 $$\varphi = \frac{{f_{1} h}}{{2(1 + \alpha \varepsilon_{0} )}}\left( {\frac{{\partial^{2} w}}{{\partial r^{2} }} + \frac{\partial w}{{r\partial r}}} \right)\left( {\frac{2}{h}z - \frac{{e^{\lambda z} - e^{ - \lambda z} }}{{e^{\lambda h/2} - e^{ - \lambda h/2} }}} \right) + \frac{{V_{0} }}{h}z + \frac{{V_{0} }}{2}.$$

The governing equation is written as

32 $$\frac{{c_{7} h^{3} }}{6}\nabla^{6} w - \left( {\frac{{c_{4} h^{3} }}{6} + 2c_{1} h - d_{2} } \right)\nabla^{4} w - \rho h\ddot{w} + q = 0.$$

And the mechanical boundary conditions are

33 $$\begin{aligned} & S_{w}^{L} (R)\delta w(R) - S_{w}^{L} (0)\delta w(0) = 0 \\ & S_{w1}^{L} (R)\delta w_{,r} (R) - S_{w1}^{L} (0)\delta w_{,r} (0) = 0 \\ & S_{w2}^{L} (R)\delta w_{,rr} (R) - S_{w2}^{L} (0)\delta w_{,rr} (0) = 0, \\ \end{aligned}$$

where

34 $$\begin{aligned} S_{w}^{L} (r) & = r\left[ {\frac{{c_{7} h^{3} }}{6}\left( {\frac{{\partial^{5} w}}{{\partial r^{5} }} + 2\frac{{\partial^{4} w}}{{r\partial r^{4} }} - 3\frac{{\partial^{3} w}}{{r^{2} \partial r^{3} }} + 3\frac{{\partial^{2} w}}{{r^{3} \partial r^{2} }} - 3\frac{\partial w}{{r^{4} \partial r}}} \right) - \left( {2c_{1} h + \frac{{c_{4} h^{3} }}{6} - d_{2} } \right)\left( {\frac{{\partial^{3} w}}{{\partial r^{3} }} + \frac{{\partial^{2} w}}{{r\partial r^{2} }} - \frac{\partial w}{{r^{2} \partial r}}} \right)} \right] \\ S_{w1}^{L} (r) & = r\left[ { - \frac{{c_{7} h^{3} }}{6}\left( {\frac{{\partial^{4} w}}{{\partial r^{4} }} + \frac{{\partial^{3} w}}{{r\partial r^{3} }}} \right) + \frac{{(2c_{8} + c_{9} )h^{3} }}{4}\left( {\frac{{\partial^{2} w}}{{r^{2} \partial r^{2} }} - \frac{\partial w}{{r^{3} \partial r}}} \right) + \left( {2c_{1} h + \frac{{c_{4} h^{3} }}{6} - d_{2} } \right)\frac{{\partial^{2} w}}{{\partial r^{2} }}} \right. \\ & \quad \left. { + \left( {c_{3} h + \frac{{c_{5} h^{3} }}{6} - d_{2} } \right)\frac{\partial w}{{r\partial r}} + \frac{{f_{1} }}{\alpha }V_{0} } \right] \\ S_{w2}^{L} (r) & = r\left[ {\frac{{c_{7} h^{3} }}{6}\frac{{\partial^{3} w}}{{\partial r^{3} }} + \frac{{c_{9} h^{3} }}{4}\left( {\frac{{\partial^{2} w}}{{r\partial r^{2} }} - \frac{\partial w}{{r^{2} \partial r}}} \right)} \right]. \\ \end{aligned}$$

The present nonlinear model of flexoelectric circular plate has been given in Eqs. (24)–(29) which can be further converted into proper dimensionless indexes. By introducing dimensionless parameters,

35 $$\xi = \frac{r}{R},\overline{w} = \frac{w}{h},\overline{u} = \frac{u}{h},\vartheta = \frac{h}{R},\overline{q}_{0} = \frac{q}{{\vartheta^{4} (c_{4} - d_{1} )}},\varsigma = \sqrt {\frac{{c_{4} - d_{1} }}{{\rho R^{2} }}} t,\overline{V}_{0} = \frac{{R^{2} f_{1} V_{0} }}{{\alpha h^{4} (c_{4} - d_{1} )}}$$

the governing equations are normalized as

36 $$\begin{aligned} & 2\frac{{\overline{u}}}{{\xi^{2} }} - 2\frac{{\partial \overline{u}}}{\xi \partial \xi } - 2\frac{{\partial^{2} \overline{u}}}{{\partial \xi^{2} }} - \frac{{\vartheta b_{0} }}{\xi }\left( {\frac{{\partial \overline{w}}}{\partial \xi }} \right)^{2} + \vartheta b_{2} \left( {\frac{{\partial \overline{w}}}{{\xi^{2} \partial \xi }}\frac{{\partial^{2} \overline{w}}}{{\partial \xi^{2} }} - \frac{1}{\xi }\left( {\frac{{\partial^{2} \overline{w}}}{{\partial \xi^{2} }}} \right)^{2} - \frac{{\partial \overline{w}}}{\xi \partial \xi }\frac{{\partial^{3} \overline{w}}}{{\partial \xi^{3} }}} \right) \\ & \quad - 2\vartheta \frac{{\partial \overline{w}}}{\partial \xi }\frac{{\partial^{2} \overline{w}}}{{\partial \xi^{2} }} + \frac{{\vartheta b_{1} }}{\xi }\left( {\frac{{\partial \overline{w}}}{\partial \xi }} \right)^{2} + 2b_{3} \left( {\frac{{\partial^{4} \overline{u}}}{{\partial \xi^{4} }} + 2\frac{{\partial^{3} \overline{u}}}{{\xi \partial \xi^{3} }} - 3\frac{{\partial^{2} \overline{u}}}{{\xi^{2} \partial \xi^{2} }} + 3\frac{{\partial \overline{u}}}{{\xi^{3} \partial \xi }} - 3\frac{{\overline{u}}}{{\xi^{4} }}} \right) \\ & \quad + 2\vartheta b_{3} \left( {\frac{2}{\xi }\left( {\frac{{\partial^{2} \overline{w}}}{{\partial \xi^{2} }}} \right)^{2} + \frac{{\partial \overline{w}}}{\partial \xi }\frac{{\partial^{4} \overline{w}}}{{\partial \xi^{4} }} + 3\frac{{\partial^{3} \overline{w}}}{{\partial \xi^{3} }}\frac{{\partial^{2} \overline{w}}}{{\partial \xi^{2} }} + 2\frac{{\partial \overline{w}}}{\partial \xi }\frac{{\partial^{3} \overline{w}}}{{\xi \partial \xi^{3} }} - 2\frac{{\partial \overline{w}}}{\partial \xi }\frac{{\partial^{2} \overline{w}}}{{\xi^{2} \partial \xi^{2} }}} \right) + \frac{{\partial^{2} \overline{u}}}{{\partial \varsigma^{2} }} = 0, \\ \end{aligned}$$

37 $$\begin{aligned} & \frac{{b_{3} }}{6}\left( {\frac{{\partial^{6} \overline{w}}}{{\partial \xi^{6} }} + 3\frac{{\partial^{5} \overline{w}}}{{\xi \partial \xi^{5} }} - 3\frac{{\partial^{4} \overline{w}}}{{\xi^{2} \partial \xi^{4} }} + 6\frac{{\partial^{3} \overline{w}}}{{\xi^{3} \partial \xi^{3} }} - 9\frac{{\partial^{2} \overline{w}}}{{\xi^{4} \partial \xi^{2} }} + 9\frac{{\partial \overline{w}}}{{\xi^{5} \partial \xi }}} \right) - b_{4} \left( {\frac{{\partial^{4} \overline{w}}}{{\partial \xi^{4} }} + 2\frac{{\partial^{3} \overline{w}}}{{\xi \partial \xi^{3} }} - \frac{{\partial^{2} \overline{w}}}{{\xi^{2} \partial \xi^{2} }} + \frac{{\partial \overline{w}}}{{\xi^{3} \partial \xi }}} \right) \\ & \quad + \frac{2}{\vartheta }\left[ {\frac{{\partial^{2} \overline{u}}}{{\partial \xi^{2} }}\frac{{\partial \overline{w}}}{\partial \xi } + \frac{{\partial \overline{u}}}{\partial \xi }\left( {\frac{{\partial^{2} \overline{w}}}{{\partial \xi^{2} }} + \frac{{\partial \overline{w}}}{\xi \partial \xi }} \right)} \right] + \left( {\frac{{\partial \overline{w}}}{\partial \xi }} \right)^{2} \left( {3\frac{{\partial^{2} \overline{w}}}{{\partial \xi^{2} }} + \frac{{\partial \overline{w}}}{\xi \partial \xi }} \right) + \frac{{2b_{9} }}{\vartheta }\left( {\frac{{\partial \overline{u}}}{\partial \xi }\frac{{\partial \overline{w}}}{\xi \partial \xi } + \frac{{\overline{u}}}{\xi }\frac{{\partial^{2} \overline{w}}}{{\partial \xi^{2} }}} \right) \\ & \quad - 4b_{5} \left( {\frac{{\partial \overline{w}}}{\xi \partial \xi }} \right)^{2} \left( {\frac{{\partial \overline{w}}}{\xi \partial \xi } - 3\frac{{\partial^{2} \overline{w}}}{{\partial \xi^{2} }}} \right) - \frac{{{2}b_{3} }}{\vartheta }\left[ {\frac{{\partial^{4} \overline{u}}}{{\partial \xi^{4} }}\frac{{\partial \overline{w}}}{\partial \xi } + \frac{{\partial^{3} \overline{u}}}{{\partial \xi^{3} }}\left( {\frac{{\partial^{2} \overline{w}}}{{\partial \xi^{2} }} + 2\frac{{\partial \overline{w}}}{\xi \partial \xi }} \right) + \frac{{\partial^{2} \overline{u}}}{{\partial \xi^{2} }}\frac{{\partial^{2} \overline{w}}}{{\xi \partial \xi^{2} }}} \right] \\ & \quad - {2}b_{3} \left[ {3\frac{{\partial \overline{w}}}{\xi \partial \xi }\left( {\frac{{\partial^{2} \overline{w}}}{{\partial \xi^{2} }}} \right)^{2} + 2\left( {\frac{{\partial \overline{w}}}{\partial \xi }} \right)^{2} \frac{{\partial^{3} \overline{w}}}{{\xi \partial \xi^{3} }} + \left( {\frac{{\partial \overline{w}}}{\partial \xi }} \right)^{2} \frac{{\partial^{4} \overline{w}}}{{\partial \xi^{4} }} + \left( {\frac{{\partial^{2} \overline{w}}}{{\partial \xi^{2} }}} \right)^{3} + 4\frac{{\partial \overline{w}}}{\partial \xi }\frac{{\partial^{2} \overline{w}}}{{\partial \xi^{2} }}\frac{{\partial^{3} \overline{w}}}{{\partial \xi^{3} }}} \right] \\ & \quad + 4\frac{{b_{6} }}{\vartheta }\left[ {\frac{{\partial^{2} \overline{u}}}{{\xi^{2} \partial \xi^{2} }}\frac{{\partial \overline{w}}}{\partial \xi } + \left( {\frac{{\partial \overline{u}}}{{\xi^{2} \partial \xi }} - \frac{{\overline{u}}}{{\xi^{3} }}} \right)\left( {\frac{{\partial^{2} \overline{w}}}{{\partial \xi^{2} }} - 2\frac{{\partial \overline{w}}}{\xi \partial \xi }} \right)} \right] + \frac{{b_{2} }}{\vartheta }\left[ { - \frac{{\partial^{3} \overline{u}}}{{\partial \xi^{3} }}\frac{{\partial \overline{w}}}{\xi \partial \xi } - \frac{{\partial^{2} \overline{u}}}{{\xi \partial \xi^{2} }}\left( {\frac{{\partial^{2} \overline{w}}}{{\partial \xi^{2} }} - 3\frac{{\partial \overline{w}}}{\xi \partial \xi }} \right)} \right. \\ & \quad \left. { + 3\left( {\frac{{\partial \overline{u}}}{{\xi^{2} \partial \xi }} - \frac{{\overline{u}}}{{\xi^{3} }}} \right)\left( {\frac{{\partial^{2} \overline{w}}}{{\partial \xi^{2} }} - 2\frac{{\partial \overline{w}}}{\xi \partial \xi }} \right)} \right] - \frac{1}{{\vartheta^{2} }}\frac{{\partial^{2} \overline{w}}}{{\partial \varsigma^{2} }} + \overline{q}_{0} = 0 \\ \end{aligned}$$

the dimensionless initial conditions are

38 $$\dot{\overline{u}}\delta \overline{u}|_{{\varsigma_{1} }}^{{\varsigma_{2} }} = 0,\dot{\overline{w}}\delta \overline{w}|_{{\varsigma_{1} }}^{{\varsigma_{2} }} = 0,$$

and the boundary conditions are reformulated as

39 $$\begin{aligned} & \overline{S}_{u} (1)\delta \overline{u}(1) - \overline{S}_{u} (0)\delta \overline{u}(0) = 0,\overline{S}_{u1} (1)\delta \overline{u}_{,\xi } (1) - \overline{S}_{u1} (0)\delta \overline{u}_{,\xi } (0) = 0 \\ & \overline{S}_{w} (1)\delta \overline{w}(1) - \overline{S}_{w} (0)\delta \overline{w}(0) = 0,\overline{S}_{w1} (1)\delta \overline{w}_{,\xi } (1) - \overline{S}_{w1} (0)\delta \overline{w}_{,\xi } (0) = 0 \\ & \overline{S}_{w2} (1)\delta \overline{w}_{,\xi \xi } (1) - \overline{S}_{w2} (0)\delta \overline{w}_{,\xi \xi } (0) = 0, \\ \end{aligned}$$

where the parameters *b*_*i*_(*i* = 1 ~ 9) are given by

40 $$\begin{aligned} & b_{0} = \frac{{c_{4} }}{{c_{4} - d_{1} }},b_{1} = \frac{{c_{5} }}{{c_{4} - d_{1} }},b_{2} = \frac{{c_{9} }}{{R^{2} (c_{4} - d_{1} )}},b_{3} = \frac{{c_{7} }}{{R^{2} (c_{4} - d_{1} )}},b_{4} = \frac{{c_{4} h^{3} + 12c_{1} h - 6d_{2} }}{{6h^{3} (c_{4} - d_{1} )}} \\ & b_{5} = \frac{{c_{2} }}{{R^{2} (c_{4} - d_{1} )}}, b_{6} = \frac{{c_{8} }}{{R^{2} (c_{4} - d_{1} )}}, b_{7} = \frac{{c_{9} }}{{2c_{7} }},b_{8} = \frac{{c_{3} h - d_{2} }}{{h^{3} (c_{4} - d_{1} )}},b_{9} = \frac{{c_{5} - d_{1} }}{{c_{4} - d_{1} }} \\ \end{aligned}$$

and the dimensionless quantities $\overline{S}_{u}$, $\overline{S}_{u1}$, $\overline{S}_{w}$, $\overline{S}_{w1}$, $\overline{S}_{w2}$ are given in Appendix B.

## Nonlinear bending of flexoelectric circular plate

The differential quadrature method (DQM)^42–44^ can be used to solve the nonlinear governing equations (36) and (37) with the corresponding boundary conditions for the axisymmetric bending of flexoelectric circular plate. For a function *f*(*x*), its *k*-order partial derivatives with respect to *x* at any sample point can be written as

41 $$\frac{{\partial^{k} f(x_{i} )}}{{\partial x^{k} }} = \sum\limits_{j = 1}^{N} {A_{ij}^{(k)} f(x_{j} )}$$

with *N* denoting the total number of discrete points and $A_{ij}^{(k)}$ being the *k*-order weighting coefficients matrix whose recursion formula can be found in Li et al.^41^. The normalized Gauss–Chebyshev–Lobatto points are used to generate the DQM point system,

42 $$\xi (i) = \frac{1}{2}\left[ {1 - \cos \left( {\frac{i - 1}{{N - 1}}\pi } \right)} \right],\quad i = 1,2, \ldots ,N.$$

By applying DQM, the discretized equilibrium equations are given as

43 $$\begin{aligned} & \left( {\frac{2}{{\xi_{i}^{2} }} - \frac{{6b_{3} }}{{\xi_{i}^{4} }}} \right)\overline{u}_{i} + \left( {\frac{{6b_{3} }}{{\xi_{i}^{3} }} - \frac{2}{{\xi_{i} }}} \right)\sum\limits_{j = 1}^{N} {A_{ij}^{(1)} \overline{u}_{j} } - \left( {2 + \frac{{6b_{3} }}{{\xi_{i}^{2} }}} \right)\sum\limits_{j = 1}^{N} {A_{ij}^{(2)} \overline{u}_{j} } + \frac{{4b_{3} }}{{\xi_{i} }}\sum\limits_{j = 1}^{N} {A_{ij}^{(3)} \overline{u}_{j} } + 2b_{3} \sum\limits_{j = 1}^{N} {A_{ij}^{(4)} \overline{u}_{j} } \\ & \quad + \left( {\frac{{\vartheta b_{1} }}{{\xi_{i} }} - \frac{{\vartheta b_{0} }}{{\xi_{i} }}} \right)\sum\limits_{j = 1}^{N} {A_{ij}^{(1)} \overline{w}_{j} } \sum\limits_{j = 1}^{N} {A_{ij}^{(1)} \overline{w}_{j} } + \left( {\frac{{\vartheta b_{2} }}{{\xi_{i}^{2} }} - \frac{{4\vartheta b_{3} }}{{\xi_{i}^{2} }} - 2\vartheta } \right)\sum\limits_{j = 1}^{N} {A_{ij}^{(1)} \overline{w}_{j} } \sum\limits_{j = 1}^{N} {A_{ij}^{(2)} \overline{w}_{j} } \\ & \quad + \left( {\frac{{4\vartheta b_{3} }}{{\xi_{i} }} - \frac{{\vartheta b_{2} }}{{\xi_{i} }}} \right)\sum\limits_{j = 1}^{N} {A_{ij}^{(2)} \overline{w}_{j} } \sum\limits_{j = 1}^{N} {A_{ij}^{(2)} \overline{w}_{j} } + \left( {\frac{{4\vartheta b_{3} }}{{\xi_{i} }} - \frac{{\vartheta b_{2} }}{{\xi_{i} }}} \right)\sum\limits_{j = 1}^{N} {A_{ij}^{(1)} \overline{w}_{j} } \sum\limits_{j = 1}^{N} {A_{ij}^{(3)} \overline{w}_{j} } \\ & \quad + 2\vartheta b_{3} \sum\limits_{j = 1}^{N} {A_{ij}^{(1)} \overline{w}_{j} } \sum\limits_{j = 1}^{N} {A_{ij}^{(4)} \overline{w}_{j} } + 6\vartheta b_{3} \sum\limits_{j = 1}^{N} {A_{ij}^{(2)} \overline{w}_{j} } \sum\limits_{j = 1}^{N} {A_{ij}^{(3)} \overline{w}_{j} } + \frac{{\partial^{2} \overline{u}_{i} }}{{\partial \varsigma^{2} }} = 0, \\ \end{aligned}$$

44 $$\begin{aligned} & \frac{{b_{3} }}{6}\sum\limits_{j = 1}^{N} {A_{ij}^{(6)} \overline{w}_{j} } + \frac{1}{2}\frac{{b_{3} }}{{\xi_{i} }}\sum\limits_{j = 1}^{N} {A_{ij}^{(5)} \overline{w}_{j} } - \left( {\frac{1}{2}\frac{{b_{3} }}{{\xi_{i}^{2} }} + b_{4} } \right)\sum\limits_{j = 1}^{N} {A_{ij}^{(4)} \overline{w}_{j} } + \left( {\frac{{b_{3} }}{{\xi_{i}^{3} }} - \frac{{2b_{4} }}{{\xi_{i} }}} \right)\sum\limits_{j = 1}^{N} {A_{ij}^{(3)} \overline{w}_{j} } \\ & \quad + \left( {\frac{{b_{4} }}{{\xi_{i}^{2} }} - \frac{3}{2}\frac{{b_{3} }}{{\xi_{i}^{4} }}} \right)\sum\limits_{j = 1}^{N} {A_{ij}^{(2)} \overline{w}_{j} } + \left( {\frac{3}{2}\frac{{b_{3} }}{{\xi_{i}^{5} }} - \frac{{b_{4} }}{{\xi_{i}^{3} }}} \right)\sum\limits_{j = 1}^{N} {A_{ij}^{(1)} \overline{w}_{j} } + \left( {\frac{1}{{\xi_{i} }} - \frac{{4b_{5} }}{{\xi_{i}^{3} }}} \right)\sum\limits_{j = 1}^{N} {A_{ij}^{(1)} \overline{w}_{j} } \sum\limits_{j = 1}^{N} {A_{ij}^{(1)} \overline{w}_{j} } \sum\limits_{j = 1}^{N} {A_{ij}^{(1)} \overline{w}_{j} } \\ & \quad + \left( {3 + \frac{{12b_{5} }}{{\xi_{i}^{2} }}} \right)\sum\limits_{j = 1}^{N} {A_{ij}^{(1)} \overline{w}_{j} } \sum\limits_{j = 1}^{N} {A_{ij}^{(1)} \overline{w}_{j} } \sum\limits_{j = 1}^{N} {A_{ij}^{(2)} \overline{w}_{j} } - \frac{{6b_{3} }}{{\xi_{i} }}\sum\limits_{j = 1}^{N} {A_{ij}^{(1)} \overline{w}_{j} } \sum\limits_{j = 1}^{N} {A_{ij}^{(2)} \overline{w}_{j} } \sum\limits_{j = 1}^{N} {A_{ij}^{(2)} \overline{w}_{j} } \\ & \quad - \frac{{4b_{3} }}{{\xi_{i} }}\sum\limits_{j = 1}^{N} {A_{ij}^{(1)} \overline{w}_{j} } \sum\limits_{j = 1}^{N} {A_{ij}^{(1)} \overline{w}_{j} } \sum\limits_{j = 1}^{N} {A_{ij}^{(3)} \overline{w}_{j} } - 2b_{3} \sum\limits_{j = 1}^{N} {A_{ij}^{(1)} \overline{w}_{j} } \sum\limits_{j = 1}^{N} {A_{ij}^{(1)} \overline{w}_{j} } \sum\limits_{j = 1}^{N} {A_{ij}^{(4)} \overline{w}_{j} } \\ & \quad - 2b_{3} \sum\limits_{j = 1}^{N} {A_{ij}^{(2)} \overline{w}_{j} } \sum\limits_{j = 1}^{N} {A_{ij}^{(2)} \overline{w}_{j} } \sum\limits_{j = 1}^{N} {A_{ij}^{(2)} \overline{w}_{j} } - 8b_{3} \sum\limits_{j = 1}^{N} {A_{ij}^{(1)} \overline{w}_{j} } \sum\limits_{j = 1}^{N} {A_{ij}^{(2)} \overline{w}_{j} } \sum\limits_{j = 1}^{N} {A_{ij}^{(3)} \overline{w}_{j} } \\ & \quad + \frac{{2(3b_{2} + 4b_{6} )}}{\vartheta }\frac{{\overline{u}_{i} }}{{\xi_{i}^{4} }}\sum\limits_{j = 1}^{N} {A_{ij}^{(1)} \overline{w}_{j} } + \left( {2b_{9} - \frac{{3b_{2} + 4b_{6} }}{{\xi_{i}^{2} }}} \right)\frac{{\overline{u}_{i} }}{{\vartheta \xi_{i} }}\sum\limits_{j = 1}^{N} {A_{ij}^{(2)} \overline{w}_{j} } \\ & \quad + \frac{2}{{\vartheta \xi_{i} }}\left( {1 + b_{9} - \frac{{3b_{2} + 4b_{6} }}{{\xi_{i}^{2} }}} \right)\sum\limits_{j = 1}^{N} {A_{ij}^{(1)} \overline{u}_{j} } \sum\limits_{j = 1}^{N} {A_{ij}^{(1)} \overline{w}_{j} } - \frac{{{2}b_{3} + b_{2} }}{{\vartheta \xi_{i} }}\sum\limits_{j = 1}^{N} {A_{ij}^{(2)} \overline{u}_{j} } \sum\limits_{j = 1}^{N} {A_{ij}^{(2)} \overline{w}_{j} } \\ & \quad + \frac{1}{\vartheta }\left( {\frac{{3b_{2} + 4b_{6} }}{{\xi_{i}^{2} }} + 2} \right)\left( {\sum\limits_{j = 1}^{N} {A_{ij}^{(1)} \overline{u}_{j} } \sum\limits_{j = 1}^{N} {A_{ij}^{(2)} \overline{w}_{j} } + \sum\limits_{j = 1}^{N} {A_{ij}^{(2)} \overline{u}_{j} } \sum\limits_{j = 1}^{N} {A_{ij}^{(1)} \overline{w}_{j} } } \right) \\ & \quad - \frac{{4b_{3} + b_{2} }}{{\vartheta \xi_{i} }}\sum\limits_{j = 1}^{N} {A_{ij}^{(3)} \overline{u}_{j} } \sum\limits_{j = 1}^{N} {A_{ij}^{(1)} \overline{w}_{j} } - \frac{{{2}b_{3} }}{\vartheta }\sum\limits_{j = 1}^{N} {A_{ij}^{(4)} \overline{u}_{j} } \sum\limits_{j = 1}^{N} {A_{ij}^{(1)} \overline{w}_{j} } - \frac{{{2}b_{3} }}{\vartheta }\sum\limits_{j = 1}^{N} {A_{ij}^{(3)} \overline{u}_{j} } \sum\limits_{j = 1}^{N} {A_{ij}^{(2)} \overline{w}_{j} } - \frac{1}{{\vartheta^{2} }}\frac{{\partial^{2} \overline{w}_{i} }}{{\partial \varsigma^{2} }} = - \overline{q}_{0} \\ \end{aligned}$$

And the discretized dimensionless quantities $\overline{S}_{u}$, $\overline{S}_{u1}$, $\overline{S}_{w}$, $\overline{S}_{w1}$, $\overline{S}_{w2}$ of the boundary conditions Eq. (39) are given in Appendix Eqs. (B.11)–(B.15), respectively.

By denoting the displacement vector as

45 $${\mathbf{d}} = \{ \{ \overline{u}_{i} \}^{T} ,\{ \overline{w}_{i} \}^{T} \}^{T} ,\quad i = 1,2, \ldots ,N.$$

Then the discrete governing equations Eqs. (43) and (44), together with the boundary conditions, can be written in matrix form

46 $$({\mathbf{K}}_{{\mathbf{L}}} + {\mathbf{K}}_{{{\mathbf{NL}}}} ){\mathbf{d}} + {\mathbf{M\ddot{d}}} = {\mathbf{F}}$$

in which **K**_**L**_ and **K**_**NL**_ represent equivalent linear and nonlinear stiffness matrices, **M** denotes the mass matrix and **F** denotes the external force vector.

### Solution of simply supported circular plate

For the nonlinear static bending of axisymmetric flexoelectric circular plate subjected to a uniformly distributed load *q* and a constant electric voltage *V*_0_ between its upper and lower surfaces, the corresponding discrete equilibrium equations and boundary conditions simplified from Eq. (46) are given by

47 $$({\mathbf{K}}_{{\mathbf{L}}} + {\mathbf{K}}_{{{\mathbf{NL}}}} ){\mathbf{d}} = {\mathbf{F}}.$$

For the simply supported axisymmetric circular plate, its boundary conditions are

48 $$\overline{u}(\xi_{N} ) = 0,\overline{w}(\xi_{N} ) = 0,\overline{S}_{u1} (\xi_{N} ) = 0,\overline{S}_{w1} (\xi_{N} ) = 0,\overline{S}_{w2} (\xi_{N} ) = 0.$$

And the regularity conditions are listed as

49 $$\overline{u}(\xi_{1} ) = 0,\overline{w}_{,\xi } (\xi_{1} ) = 0.$$

These nonlinear equations can be solved according to Newton–Raphson method. Of course, the linear axisymmetric bending of flexoelectric circular plate can also be solved from Eqs. (32)–(34) by DQM. In addition, as a contrast, the theoretical solution of linear axisymmetric bending is derived as^41^

50 $$\overline{w} = a_{1} + a_{2} \xi^{2} + a_{5} I_{0} (s\xi ) + \frac{{\overline{q}_{0} \xi^{4} }}{{64b_{4} }},$$

where the underdetermined coefficients are given by

51 $$a_{1} { = }\frac{{\overline{q}_{0} }}{{8b_{4} }}\left[ {\frac{{(b_{7} + 1)I_{0} (s)}}{{g_{1} }} - \frac{1 + 8p}{8}} \right],a_{2} = \frac{{\overline{q}_{0} p}}{{8b_{4} }},a_{5} = \overline{ - }\frac{{\overline{q}_{0} (b_{7} + 1)}}{{8b_{4} g_{1} }},$$

where the coefficients *s*, *p*, *g*_1_ are given by

52 $$\begin{aligned} & s = \sqrt {\frac{{6b_{4} }}{{b_{3} }}} ,p = \frac{3}{{b_{1} + 6b_{4} + 6b_{8} }}\left( {\frac{{g_{3} }}{2}{ + }\frac{{(2b_{7} + 1)g_{2} }}{{g_{1} }}} \right) \\ & g_{1} = \frac{{b_{7} s^{2} }}{2}(I_{0} (s) + I_{2} (s)) + s\left( {\frac{{s^{2} }}{4} - b_{7} } \right)I_{1} (s) + \frac{{s^{3} }}{12}I_{3} (s) \\ & g_{2} = \frac{{s^{2} (b_{2} + b_{4} + 2b_{6} )}}{8}I_{0} (s) + g_{0} sI_{1} (s) + \frac{{s^{2} (b_{2} + 2b_{6} )}}{8}I_{2} (s) - \frac{{b_{4} }}{4}sI_{3} (s) - \frac{{b_{3} s^{4} }}{48}I_{4} (s) \\ & g_{3} = - \frac{1}{6}b_{1} - \frac{1}{2}b_{2} + 2b_{3} - 3b_{4} - b_{6} - b_{8} - \overline{V}_{0} \frac{{16b_{4} }}{{q_{0} }} \\ & g_{0} = \frac{1}{12}(2b_{1} - 3b_{2} - 9b_{4} - 6b_{6} + 12b_{8} ) \\ \end{aligned}$$

and *I*_*k*_ (*k* = 0,1,…,4) represent the *k*-order modified Bessel functions of the first kind.

### Discussions

In order to illustrate the current solution, a polyvinylidene difluoride (PVDF) axisymmetric circular plate is considered with its properties: *E* = 3.7 GPa, *α* = 1.38 × 10^10^ N m^2^/C^2^, *ε*_0_ = 8.854 × 10^−12^C^2^/(N m^2^), *f* = − 179 N m/C, *ρ* = 1.78 × 10^3^ kg/m^3^. The radius of the circular plate is set as 10 times the thickness and the length-scale parameters are assumed to be 1 μm.

**Figure 2:**
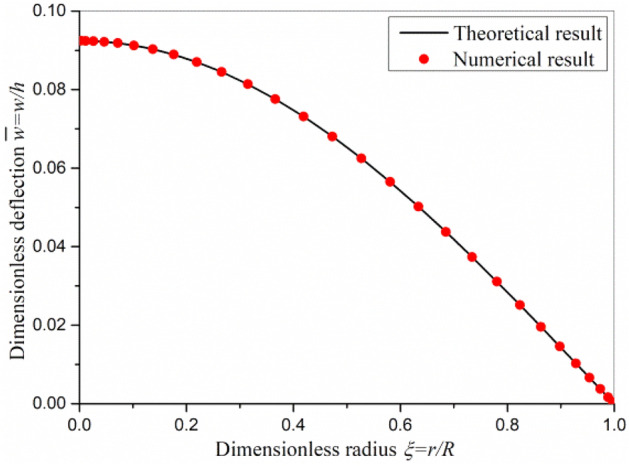
The bending deflection with *q* = 1 μN/μm^2^ and *V*_0_ = 100 V.

The dimensionless deflection is shown in Fig. 2 with the transverse load *q* being 1 μN/μm^2^ and the electric voltage *V*_0_ being 100 V. In Fig. 2, the static bending deflection of axisymmetric flexoelectric circular plate is small compared with its thickness, and the numerical results based on the linear and nonlinear models agree well with each other. This demonstrates that the accuracy of the linear model is sufficient for small deflection question. In addition, the numerical results agree well with the linear theoretical results. The DQM is suitable for solving the current problem.

**Figure 3:**
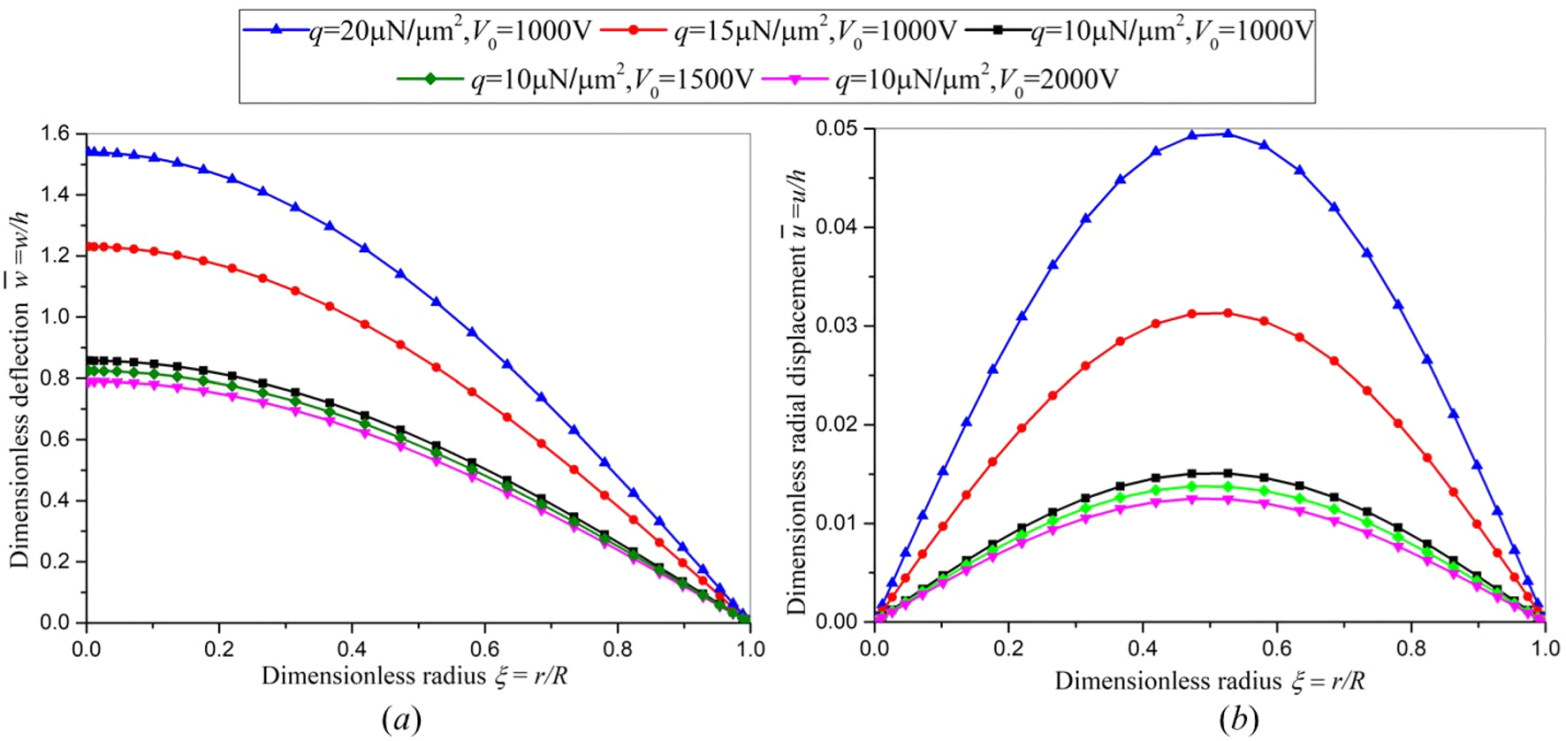
The dimensionless nonlinear displacements under different loads (**a**) the bending deflection, (**b**) the corresponding radial displacement.

**Figure 4:**
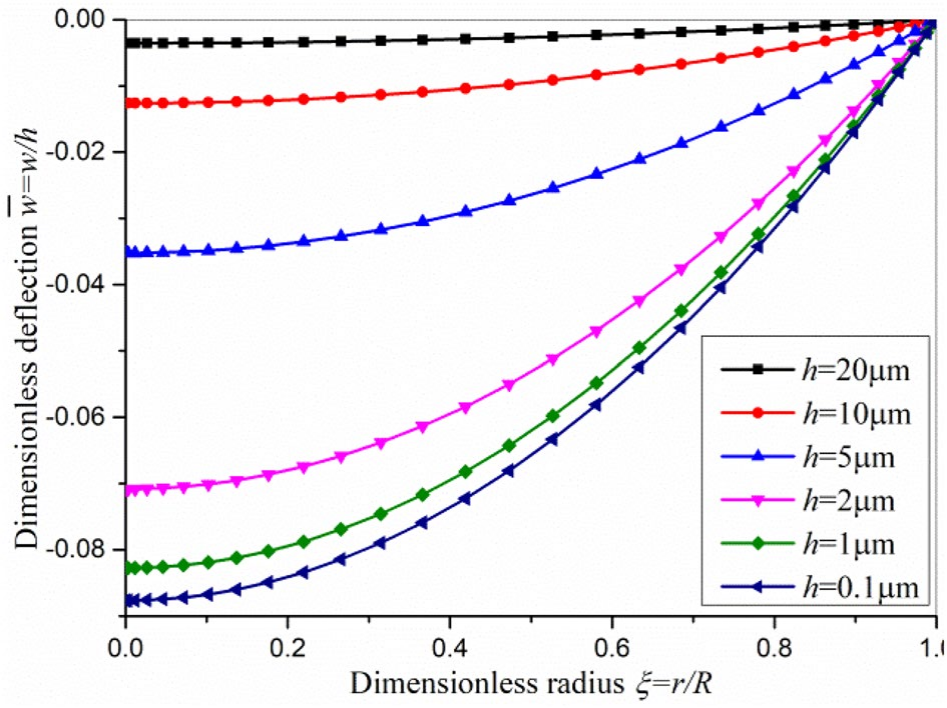
The dimensionless nonlinear bending deflections induced by inverse flexoelectric effect with *q*=0 and *V*_0_=1000 V for circular plate of different thickness.

When the static bending deflection is comparable to the thickness of plate, the linear model does not work and we have to adopt the nonlinear model. The dimensionless nonlinear displacements are shown in Fig. 3 under different loads. Figure 3 shows the maximum deflection is about 30 ~ 60 times of that of maximum radial displacement. The radial displacement is small and can be neglected compared with its lateral deflection. From Fig. 3a, it can be found that the dimensionless deflection increases with the increase of transverse load *q*, while the dimensionless deflection decreases with the increase of electric voltage *V*_0_. This is because the transverse load *q* and the electric voltage *V*_0_ generate an opposite bending deformation. The final result is a superposition of these two parts. In order to investigate the bending deflection induced by the inverse flexoelectric effect in detail, the dimensionless deflection of axisymmetric circular plate subjected to an electric voltage *V*_0_ only between the upper and lower surfaces of the plate is shown in Fig. 4. The bending deflection induced by the inverse flexoelectric effect is small and opposite in comparison with that generated by transverse load *q*. Furthermore, the inverse flexoelectric effect strengthens as the thickness of plate decreases, which can be further enhanced as the thickness of flexoelectric circular plate reaches nanometer scale.

**Figure 5:**
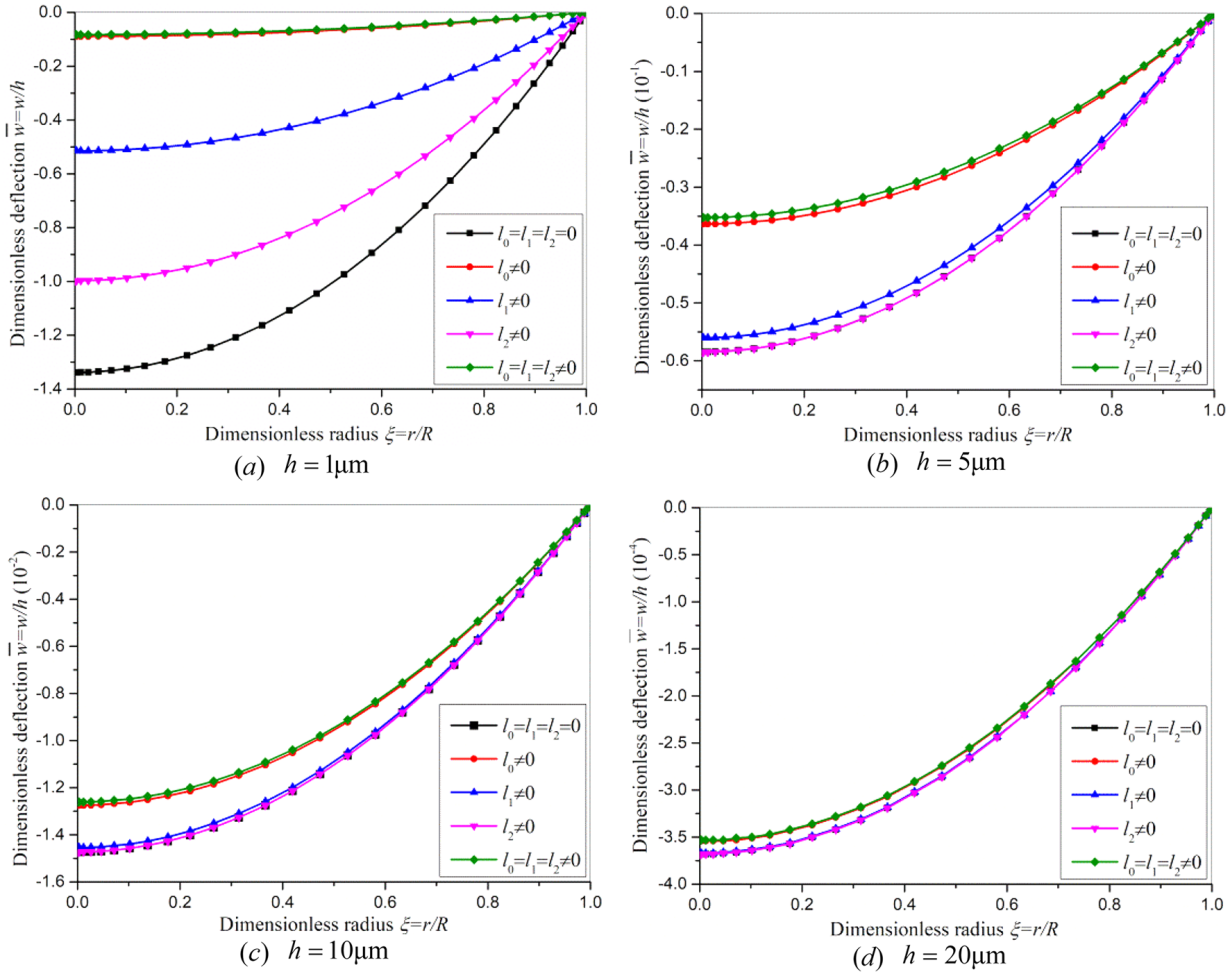
The dimensionless nonlinear bending deflections with *q*=0 and *V*_0_=1000 V (**a**) $h = 1$ μm, (**b**) $h = 5$ μm, (**c**) $h = 10$ μm, (**d**) $h = 20$ μm.

Figure 5 reveals the influence of strain gradient stiffening effect on the dimensionless bending deflection in the inverse flexoelectric process. When the plate thickness is comparable with the material length scale parameter, the dimensionless deflection with *l*_0_ = *l*_1_ = *l*_2_ ≠ 0 (including all strain gradient stiffening effect) is far smaller than that with *l*_0_ = *l*_1_ = *l*_2_ = 0 (ignoring all the strain gradient stiffening effect). This demonstrates the stiffening effect of strain gradient is remarkable. When the plate thickness is far great than the material length scale parameter, the dimensionless deflection with *l*_0_ = *l*_1_ = *l*_2_ ≠ 0 is close to that with *l*_0_ = *l*_1_ = *l*_2_ = 0. This demonstrates that the stiffening effect of strain gradient is negligible in comparison with the traditional plate bending rigidity. Moreover, the stiffening effects of the strain gradient spherical component (*l*_0_ ≠ 0), the deviatoric stretch gradient component (*l*_1_ ≠ 0), the symmetric rotation strain gradient component (*l*_2_ ≠ 0) are examined separately. Figure 5 shows that the strain gradient spherical component (*l*_0_ ≠ 0) dominates the stiffening effect, while the symmetric rotation strain gradient component (*l*_2_ ≠ 0) exhibits the least stiffening effect.

**Figure 6:**
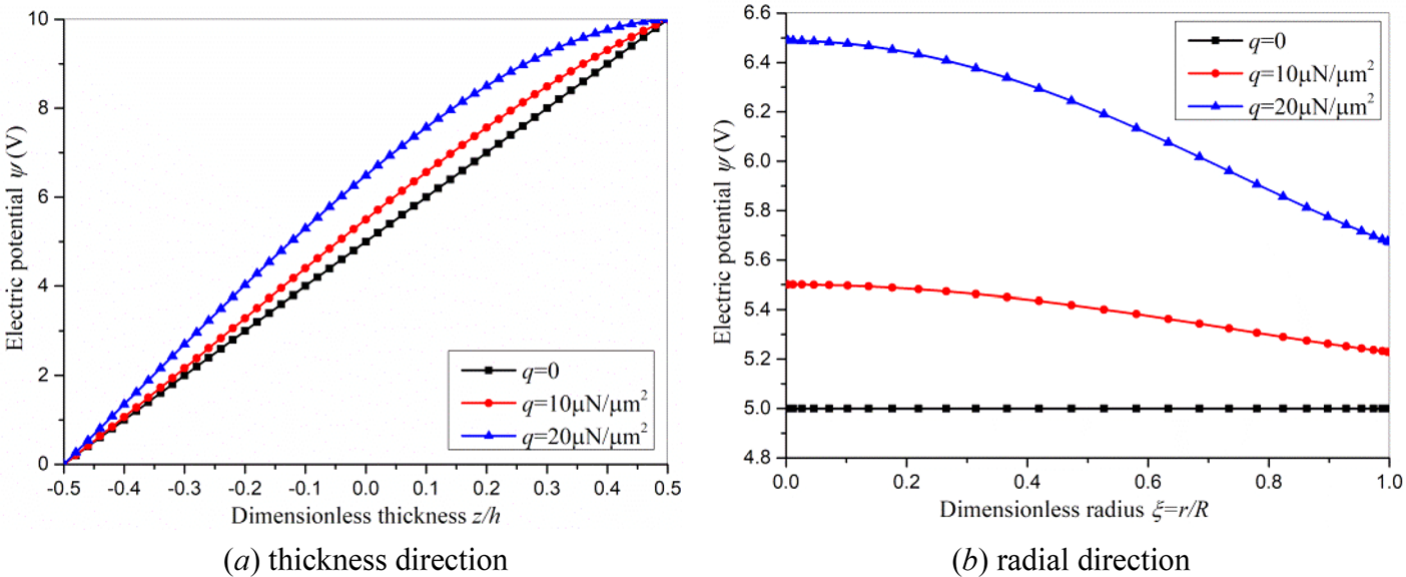
The distribution of electric potential with *V*_0_=10 V (**a**) thickness direction, (**b**) radial direction.

The distribution of electric potential is also investigated in Fig. 6. When *q* = 0, the electric potential is uniformly distributed along the thickness and is constant along the radius. In fact, the electric potential can be significantly affected by the transverse load *q*. The larger the load, the more uneven the potential distribution will be. This is because the bending deformation of circular plate under transverse load *q* can induce polarization and electric potential by direct flexoelectric effect. The greater the strain gradient, the greater the induced polarization and electric potential will be.

## Nonlinear free vibration of axisymmetric flexoelectric circular plate

From the static bending analysis of axisymmetric flexoelectric circular plate, it can be obtained from Fig. 3 that the radial displacement is small and can be neglected compared with its lateral deflection. Thus, for the nonlinear free vibration problem of axisymmetric flexoelectric circular plate, the effect of the radial displacement on the lateral vibration is neglected for simplicity and the governing equation reduced from Eq. (37) is given by

53 $$\begin{aligned} & \frac{{b_{3} }}{6}\left( {\frac{{\partial^{6} \overline{w}}}{{\partial \xi^{6} }} + 3\frac{{\partial^{5} \overline{w}}}{{\xi \partial \xi^{5} }} - 3\frac{{\partial^{4} \overline{w}}}{{\xi^{2} \partial \xi^{4} }} + 6\frac{{\partial^{3} \overline{w}}}{{\xi^{3} \partial \xi^{3} }} - 9\frac{{\partial^{2} \overline{w}}}{{\xi^{4} \partial \xi^{2} }} + 9\frac{{\partial \overline{w}}}{{\xi^{5} \partial \xi }}} \right) - b_{4} \left( {\frac{{\partial^{4} \overline{w}}}{{\partial \xi^{4} }} + 2\frac{{\partial^{3} \overline{w}}}{{\xi \partial \xi^{3} }} - \frac{{\partial^{2} \overline{w}}}{{\xi^{2} \partial \xi^{2} }} + \frac{{\partial \overline{w}}}{{\xi^{3} \partial \xi }}} \right) \\ & \quad + \left( {\frac{{\partial \overline{w}}}{\partial \xi }} \right)^{2} \left( {3\frac{{\partial^{2} \overline{w}}}{{\partial \xi^{2} }} + \frac{{\partial \overline{w}}}{\xi \partial \xi }} \right) - 4b_{5} \left( {\frac{{\partial \overline{w}}}{\xi \partial \xi }} \right)^{2} \left( {\frac{{\partial \overline{w}}}{\xi \partial \xi } - 3\frac{{\partial^{2} \overline{w}}}{{\partial \xi^{2} }}} \right) \\ & \quad - {2}b_{3} \left[ {3\frac{{\partial \overline{w}}}{\xi \partial \xi }\left( {\frac{{\partial^{2} \overline{w}}}{{\partial \xi^{2} }}} \right)^{2} + 2\left( {\frac{{\partial \overline{w}}}{\partial \xi }} \right)^{2} \frac{{\partial^{3} \overline{w}}}{{\xi \partial \xi^{3} }} + \left( {\frac{{\partial \overline{w}}}{\partial \xi }} \right)^{2} \frac{{\partial^{4} \overline{w}}}{{\partial \xi^{4} }} + \left( {\frac{{\partial^{2} \overline{w}}}{{\partial \xi^{2} }}} \right)^{3} + 4\frac{{\partial \overline{w}}}{\partial \xi }\frac{{\partial^{2} \overline{w}}}{{\partial \xi^{2} }}\frac{{\partial^{3} \overline{w}}}{{\partial \xi^{3} }}} \right] - \frac{1}{{\vartheta^{2} }}\frac{{\partial^{2} \overline{w}}}{{\partial \varsigma^{2} }} = 0. \\ \end{aligned}$$

The corresponding boundary conditions for the simply supported axisymmetric circular plate are

54 $${\text{At}}\,\xi = {1}:\overline{w}(\xi ) = 0,\overline{S}_{w1} (\xi ) = 0,\overline{S}_{w2} (\xi ) = 0.$$

The dimensionless deflection can be assumed as

55 $$\overline{w} = T(\varsigma )(1 + A_{1} \xi^{2} + A_{2} \xi^{4} + A_{3} \xi^{6} ),$$

where the coefficients *A*_1_, *A*_2_ and *A*_3_ can be determined by substituting Eq. (55) into the boundary conditions Eq. (54). Applying the Galerkin method yields

56 $$\begin{aligned} & \int_{0}^{1} {\left[ {\frac{{b_{3} }}{6}\left( {\frac{{\partial^{6} \overline{w}}}{{\partial \xi^{6} }} + 3\frac{{\partial^{5} \overline{w}}}{{\xi \partial \xi^{5} }} - 3\frac{{\partial^{4} \overline{w}}}{{\xi^{2} \partial \xi^{4} }} + 6\frac{{\partial^{3} \overline{w}}}{{\xi^{3} \partial \xi^{3} }} - 9\frac{{\partial^{2} \overline{w}}}{{\xi^{4} \partial \xi^{2} }} + 9\frac{{\partial \overline{w}}}{{\xi^{5} \partial \xi }}} \right) - b_{4} \left( {\frac{{\partial^{4} \overline{w}}}{{\partial \xi^{4} }} + 2\frac{{\partial^{3} \overline{w}}}{{\xi \partial \xi^{3} }} - \frac{{\partial^{2} \overline{w}}}{{\xi^{2} \partial \xi^{2} }} + \frac{{\partial \overline{w}}}{{\xi^{3} \partial \xi }}} \right)} \right.} \\ & \quad + \left( {\frac{{\partial \overline{w}}}{\partial \xi }} \right)^{2} \left( {3\frac{{\partial^{2} \overline{w}}}{{\partial \xi^{2} }} + \frac{{\partial \overline{w}}}{\xi \partial \xi }} \right) - 4b_{5} \left( {\frac{{\partial \overline{w}}}{\xi \partial \xi }} \right)^{2} \left( {\frac{{\partial \overline{w}}}{\xi \partial \xi } - 3\frac{{\partial^{2} \overline{w}}}{{\partial \xi^{2} }}} \right) \\ & \quad \left. { - {2}b_{3} \left[ {3\frac{{\partial \overline{w}}}{\xi \partial \xi }\left( {\frac{{\partial^{2} \overline{w}}}{{\partial \xi^{2} }}} \right)^{2} + 2\left( {\frac{{\partial \overline{w}}}{\partial \xi }} \right)^{2} \frac{{\partial^{3} \overline{w}}}{{\xi \partial \xi^{3} }} + \left( {\frac{{\partial \overline{w}}}{\partial \xi }} \right)^{2} \frac{{\partial^{4} \overline{w}}}{{\partial \xi^{4} }} + \left( {\frac{{\partial^{2} \overline{w}}}{{\partial \xi^{2} }}} \right)^{3} + 4\frac{{\partial \overline{w}}}{\partial \xi }\frac{{\partial^{2} \overline{w}}}{{\partial \xi^{2} }}\frac{{\partial^{3} \overline{w}}}{{\partial \xi^{3} }}} \right] - \frac{1}{{\vartheta^{2} }}\frac{{\partial^{2} \overline{w}}}{{\partial \varsigma^{2} }}} \right]\overline{w}\xi {\text{d}}\xi = 0. \\ \end{aligned}$$

By substituting the dimensionless deflection Eq. (55) into Eq. (56), the vibration differential equation can be derived as

57 $$\frac{{d^{2} T}}{{d\varsigma^{2} }} + \frac{{J_{1} }}{{J_{0} }}T + \frac{{J_{2} }}{{J_{0} }}T^{3} = 0$$

in which the coefficients *J*_0_, *J*_1_, *J*_2_ are

58 $$\begin{aligned} J_{0} & = \frac{1}{{\vartheta^{2} }}\int_{0}^{1} {(1 + A_{1} \xi^{2} + A_{2} \xi^{4} + A_{3} \xi^{6} )^{2} \xi d\xi } \\ J_{1} & = 64\int_{0}^{1} {(b_{4} A_{2} - 6b_{3} A_{3} + 9b_{4} A_{3} \xi^{2} )(1 + A_{1} \xi^{2} + A_{2} \xi^{4} + A_{3} \xi^{6} )\xi d\xi } \\ J_{2} & = \int_{0}^{1} {\eta (1 + A_{1} \xi^{2} + A_{2} \xi^{4} + A_{3} \xi^{6} )\xi d\xi } \\ \eta & = {16}b_{3} [(A_{1} + 6A_{2} \xi^{2} + 15A_{3} \xi^{4} )^{3} + 24\xi^{2} (A_{1} + 2A_{2} \xi^{2} + 3A_{3} \xi^{4} )^{2} (A_{2} + 10A_{3} \xi^{2} ) \\ & \quad + 3(A_{1} + 22A_{2} \xi^{2} + 95A_{3} \xi^{4} )(A_{1} + 2A_{2} \xi^{2} + 3A_{3} \xi^{4} )(A_{1} + 6A_{2} \xi^{2} + 15A_{3} \xi^{4} )] \\ & \quad - 32(A_{1} + 2A_{2} \xi^{2} + 3A_{3} \xi^{4} )^{2} [\xi^{2} (A_{1} + 5A_{2} \xi^{2} + 12A_{3} \xi^{4} ) + 2b_{5} (A_{1} + 8A_{2} \xi^{2} + 21A_{3} \xi^{4} )]. \\ \end{aligned}$$

Eq. (57) is the Duffing equation. By defining

59 $$T = \kappa \overline{T},\,\,\varpi_{0}^{2} = \frac{{J_{1} }}{{J_{0} }},\,\,\varepsilon = \frac{{J_{2} }}{{J_{1} }}\kappa^{2}$$

with *κ* and *ε* representing the small parameters, the Duffing equation can be written as

60 $$\frac{{d^{2} \overline{T}}}{{d\varsigma^{2} }} + \varpi_{0}^{2} (\overline{T} + \varepsilon \overline{T}^{3} ) = 0.$$

On the basis of the initial condition

61 $$\overline{T}(0) = \overline{B},\,\,\frac{{d\overline{T}}}{d\varsigma }|_{\varsigma = 0} = 0,$$

where $\overline{B} = B/\kappa$ and *B* denotes the amplitude, the Duffing equation Eq. (60) can be solved according to the Lindstedt–Poincaré Method^45^. The second order approximate solution is written as

62 $$\overline{T} = \overline{B}\cos (\varpi \varsigma ) - \frac{{\varepsilon \overline{B}^{3} }}{32}[\cos (\varpi \varsigma ) - \cos (3\varpi \varsigma )] + \frac{{\varepsilon^{2} \overline{B}^{5} }}{1024}[23\cos (\varpi \varsigma ) - 24\cos (3\varpi \varsigma ) + \cos (5\varpi \varsigma )],$$

where the nonlinear vibration frequency is given by

63 $$\varpi^{2} = \varpi_{0}^{2} \left( {1 + \frac{{3\varepsilon \overline{B}^{2} }}{4} - \frac{{3\varepsilon^{2} \overline{B}^{4} }}{128}} \right).$$

Finally, substituting Eqs. (59), (62), (63) into Eq. (55), the nonlinear free vibration problem of axisymmetric flexoelectric circular plate can be solved. In addition, the dimensionless natural frequency $\varpi$ is related to the natural frequency by

64 $$\varpi = \omega \sqrt {\frac{{\rho R^{2} }}{{c_{4} - d_{1} }}} .$$

**Figure 7:**
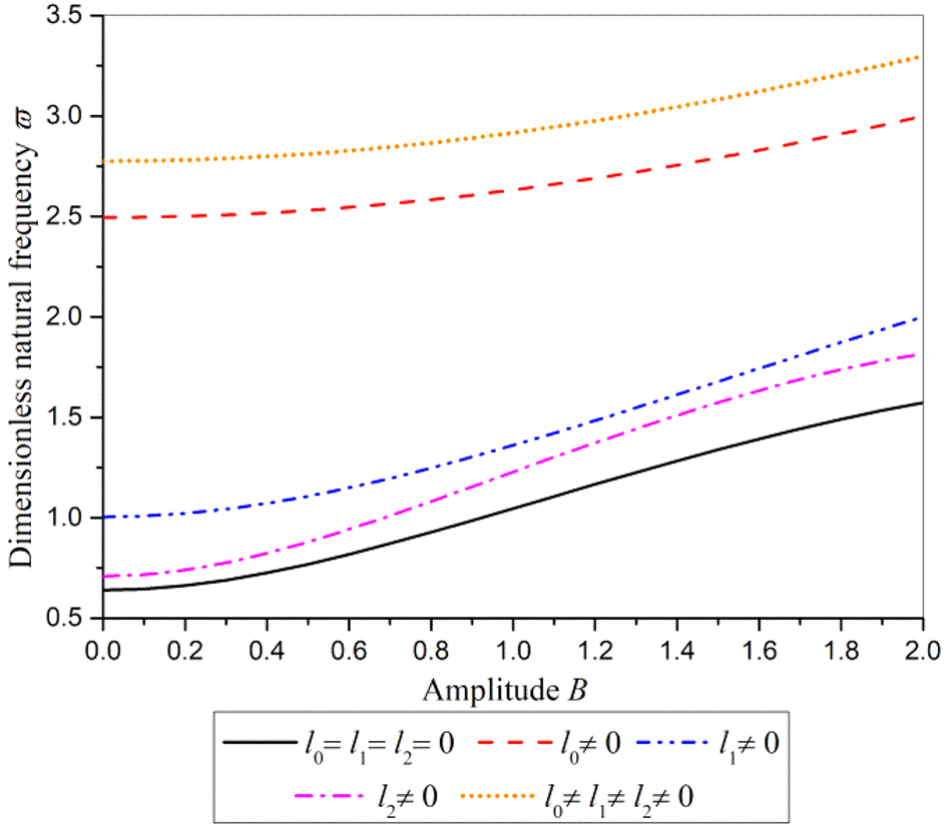
The influence of amplitude on dimensionless natural frequency.

From Eq. (63), the influence of amplitude on dimensionless natural frequency in different material length scale parameters is shown in Fig. 7. It can be found from Fig. 7 that the dimensionless natural frequency increases with the increase of amplitude. The dimensionless natural frequency with *l*_0_ = *l*_1_ = *l*_2_ ≠ 0 (including all strain gradient stiffening effect) is almost 4 times of that with *l*_0_ = *l*_1_ = *l*_2_ = 0 (ignoring all the strain gradient stiffening effect). This demonstrates the stiffening effect of strain gradient is remarkable, which is consistent with that obtained from Fig. 5. In addition, the strain gradient spherical component (*l*_0_ ≠ 0) dominates the stiffening effect, while the symmetric rotation strain gradient component (*l*_2_ ≠ 0) exhibits the least stiffening effect.

## Conclusion

In this paper, the nonlinear flexoelectric model for circular plate in large deflection deformation is established based on the flexoelectric theory. The current model will reduce to the corresponding linear model when terms associated with nonlinearity are neglected.

In order to solve the nonlinear bending equations, DQM is introduced and the validity of DQM is verified by the theoretical results of linear bending. The results demonstrate that the static bending deflection induced by the inverse flexoelectric effect is small and opposite in comparison with that generated by transverse load *q*. The final deflection is a superposition of these two parts. Although the inverse flexoelectric effect is weak, it strengthens as the thickness of plate decreases, which can be further enhanced when the plate thickness reaches nanometer scale. Moreover, the electric potential can be significantly affected by the transverse load *q*. The larger the load, the more uneven the potential distribution will be, since the bending deformation of circular plate under transverse load *q* can induce polarization and electric potential by direct flexoelectric effect.

Moreover, the nonlinear free vibration of simply supported axisymmetric flexoelectric circular plate is also investigated by combining the Galerkin method and Lindstedt–Poincaré Method. The dimensionless natural frequency increases with the increase of amplitude. When the thickness of circular plate is close to the material length scale parameter, the stiffening effect of strain gradient (mainly the strain gradient spherical component) dominates the bending rigidity compared with the traditional bending rigidity.

## Supplementary Information

Supplementary Information.
