# Supplementary material for: Nonlinear electromechanical analysis of axisymmetric thin circular plate based on flexoelectric theory
Source: Sci Rep. 2021 Nov 5;11:21762. doi: 10.1038/s41598-021-01289-0 (PMC8571295; doi:10.1038/s41598-021-01289-0)
Supplement: Supplementary file 1 — Supplementary Information. [file 41598_2021_1289_MOESM1_ESM.docx]

# Appendix A

For the nonlinear flexoelectric circular plate, by substituting the nonzero Von Kármán’s strains Eq. into Eq., the non-vanishing spherical strain gradient components are given by

$\begin{matrix} \eta_{rrr}^{V}=\frac{3}{5}[\frac{\partial^{2}u}{\partial r^{2}}+\frac{\partial u}{r\partial r}-\frac{u}{r^{2}}-z(\frac{\partial^{3}w}{\partial r^{3}}+\frac{\partial^{2}w}{r\partial r^{2}}-\frac{\partial w}{r^{2}\partial r})]+\frac{1}{5}[\frac{1}{r}(\frac{\partial w}{\partial r})^{2}+3\frac{\partial w}{\partial r}\frac{\partial^{2}w}{\partial r^{2}}] \\ \eta_{r\theta\theta}^{V}=\eta_{rzz}^{V}=\frac{1}{5}[\frac{\partial^{2}u}{\partial r^{2}}+\frac{\partial u}{r\partial r}-\frac{u}{r^{2}}-z(\frac{\partial^{3}w}{\partial r^{3}}+\frac{\partial^{2}w}{r\partial r^{2}}-\frac{\partial w}{r^{2}\partial r})-\frac{1}{2}\frac{1}{r}(\frac{\partial w}{\partial r})^{2}+\frac{\partial w}{\partial r}\frac{\partial^{2}w}{\partial r^{2}}] \\ \eta_{\theta r\theta}^{V}=\eta_{\theta\theta r}^{V}=\eta_{zrz}^{V}=\eta_{zzr}^{V}=\frac{1}{5}[\frac{\partial^{2}u}{\partial r^{2}}+\frac{\partial u}{r\partial r}-\frac{u}{r^{2}}-z(\frac{\partial^{3}w}{\partial r^{3}}+\frac{\partial^{2}w}{r\partial r^{2}}-\frac{\partial w}{r^{2}\partial r})]+\frac{1}{5}[\frac{3}{4}\frac{1}{r}(\frac{\partial w}{\partial r})^{2}+\frac{\partial w}{\partial r}\frac{\partial^{2}w}{\partial r^{2}}] \\ \eta_{zrr}^{V}=\eta_{z\theta\theta}^{V}=2\eta_{zzz}^{V}=-4\eta_{rrz}^{V}=-4\eta_{rzr}^{V}=-4\eta_{\theta\theta z}^{V}=-4\eta_{\theta z\theta}^{V}=-\frac{2}{5}(\frac{\partial^{2}w}{\partial r^{2}}+\frac{\partial w}{r\partial r}) \end{matrix}$ (A.1)

the non-vanishing components of deviatoric stretch gradient are given by

$\begin{matrix} \eta_{rrr}^{(1)}=\frac{1}{5}[2\frac{\partial^{2}u}{\partial r^{2}}-3\frac{\partial u}{r\partial r}+3\frac{u}{r^{2}}-z(2\frac{\partial^{3}w}{\partial r^{3}}-\frac{3}{r}\frac{\partial^{2}w}{\partial r^{2}}+\frac{3}{r^{2}}\frac{\partial w}{\partial r})-\frac{1}{r}(\frac{\partial w}{\partial r})^{2}+2\frac{\partial w}{\partial r}\frac{\partial^{2}w}{\partial r^{2}}] \\ \eta_{r\theta\theta}^{(1)}=\eta_{\theta r\theta}^{(1)}=\eta_{\theta\theta r}^{(1)}=\frac{1}{5}[-\frac{\partial^{2}u}{\partial r^{2}}+4\frac{\partial u}{r\partial r}-4\frac{u}{r^{2}}+z(\frac{\partial^{3}w}{\partial r^{3}}-\frac{4}{r}\frac{\partial^{2}w}{\partial r^{2}}+\frac{4}{r^{2}}\frac{\partial w}{\partial r})]+\frac{1}{15}[\frac{4}{r}(\frac{\partial w}{\partial r})^{2}-3\frac{\partial w}{\partial r}\frac{\partial^{2}w}{\partial r^{2}}] \\ \eta_{rzz}^{(1)}=\eta_{zrz}^{(1)}=\eta_{zzr}^{(1)}=\frac{1}{5}[-\frac{\partial^{2}u}{\partial r^{2}}-\frac{\partial u}{r\partial r}+\frac{u}{r^{2}}+z(\frac{\partial^{3}w}{\partial r^{3}}+\frac{\partial^{2}w}{r\partial r^{2}}-\frac{\partial w}{r^{2}\partial r})]-\frac{1}{15}[\frac{1}{r}(\frac{\partial w}{\partial r})^{2}+3\frac{\partial w}{\partial r}\frac{\partial^{2}w}{\partial r^{2}}] \\ \eta_{zzz}^{(1)}=\frac{1}{5}(\frac{\partial^{2}w}{\partial r^{2}}+\frac{\partial w}{r\partial r}), \eta_{zrr}^{(1)}=\eta_{rzr}^{(1)}=\eta_{rrz}^{(1)}=\frac{1}{15}(-4\frac{\partial^{2}w}{\partial r^{2}}+\frac{\partial w}{r\partial r}), \eta_{z\theta\theta}^{(1)}=\eta_{\theta z\theta}^{(1)}=\eta_{\theta\theta z}^{(1)}=\frac{1}{15}(\frac{\partial^{2}w}{\partial r^{2}}-4\frac{\partial w}{r\partial r}) \end{matrix}$ (A.2)

and the non-zero symmetric rotation gradient components are given by

$\begin{matrix} \eta_{r\theta\theta}^{as}=-\eta_{rzz}^{as}=-2\eta_{\theta\theta r}^{as}=-2\eta_{\theta r\theta}^{as}=2\eta_{zzr}^{as}=2\eta_{zrz}^{as}=-\frac{1}{6}\frac{1}{r}(\frac{\partial w}{\partial r})^{2} \\ \eta_{zrr}^{as}=-\eta_{z\theta\theta}^{as}=-2\eta_{rrz}^{as}=-2\eta_{rzr}^{as}=2\eta_{\theta\theta z}^{as}=2\eta_{\theta z\theta}^{as}=\frac{1}{3}(-\frac{\partial^{2}w}{\partial r^{2}}+\frac{\partial w}{r\partial r}) \end{matrix}$ (A.3)

# Appendix B

In boundary conditions Eq., the *S_u_*, *S_u_*_1_, *S_w_*, *S_w_*_1_, *S_w_*_2_ are

$\begin{matrix} S_{u}(r)=r[2h(c_{4}-d_{1})(\frac{\partial u}{\partial r}+\frac{1}{2}(\frac{\partial w}{\partial r})^{2})+2h(c_{5}-d_{1})\frac{u}{r}+2c_{8}h[3\frac{\partial u}{r^{2}\partial r}-3\frac{u}{r^{3}}+(\frac{\partial w}{r\partial r})^{2}] \\ +c_{9}h(\frac{\partial w}{r\partial r}\frac{\partial^{2}w}{\partial r^{2}}-3\frac{u}{r^{3}}+3\frac{\partial u}{r^{2}\partial r})-2c_{7}h(\frac{\partial^{2}u}{r\partial r^{2}}+\frac{\partial w}{r\partial r}\frac{\partial^{2}w}{\partial r^{2}}+\frac{\partial^{3}u}{\partial r^{3}}+(\frac{\partial^{2}w}{\partial r^{2}})^{2}+\frac{\partial w}{\partial r}\frac{\partial^{3}w}{\partial r^{3}})] \end{matrix}$ (B.1)

$S_{u1}(r)=r[2c_{7}h(\frac{\partial^{2}u}{\partial r^{2}}+\frac{\partial w}{\partial r}\frac{\partial^{2}w}{\partial r^{2}})+c_{9}h(3\frac{\partial u}{r\partial r}-3\frac{u}{r^{2}}+\frac{1}{r}(\frac{\partial w}{\partial r})^{2})]$ (B.2)

$\begin{matrix} S_{w}(r)=r[\frac{c_{7}h^{3}}{6}(\frac{\partial^{5}w}{\partial r^{5}}+2\frac{\partial^{4}w}{r\partial r^{4}}-3\frac{\partial^{3}w}{r^{2}\partial r^{3}}+3\frac{\partial^{2}w}{r^{3}\partial r^{2}}-3\frac{\partial w}{r^{4}\partial r})-[\frac{c_{4}h^{3}}{6}+2c_{1}h-d_{2}](\frac{\partial^{3}w}{\partial r^{3}}+\frac{\partial^{2}w}{r\partial r^{2}}-\frac{\partial w}{r^{2}\partial r}) \\ +h(c_{4}-d_{1})[2\frac{\partial u}{\partial r}\frac{\partial w}{\partial r}+(\frac{\partial w}{\partial r})^{3}]+2h(c_{5}-d_{1})\frac{u}{r}\frac{\partial w}{\partial r}+4c_{2}h\frac{1}{r^{2}}(\frac{\partial w}{\partial r})^{3} \\ +2c_{7}h[-\frac{\partial^{3}u}{\partial r^{3}}\frac{\partial w}{\partial r}-\frac{\partial^{2}u}{\partial r^{2}}\frac{\partial w}{r\partial r}-\frac{\partial w}{\partial r}(\frac{\partial^{2}w}{\partial r^{2}})^{2}-(\frac{\partial w}{\partial r})^{2}\frac{\partial^{2}w}{r\partial r^{2}}-(\frac{\partial w}{\partial r})^{2}\frac{\partial^{3}w}{\partial r^{3}}] \\ +4c_{8}h(\frac{\partial u}{r^{2}\partial r}\frac{\partial w}{\partial r}-\frac{u}{r^{3}}\frac{\partial w}{\partial r})+c_{9}h(-\frac{\partial^{2}u}{\partial r^{2}}\frac{\partial w}{r\partial r}+3\frac{\partial u}{r^{2}\partial r}\frac{\partial w}{\partial r}-3\frac{u}{r^{3}}\frac{\partial w}{\partial r}) \end{matrix}$ (B.3)

$\begin{matrix} S_{w1}(r)=r[(2c_{1}h+\frac{c_{4}h^{3}}{6}-d_{2})\frac{\partial^{2}w}{\partial r^{2}}+(c_{3}h-d_{2})\frac{\partial w}{r\partial r}+2c_{7}h[\frac{\partial^{2}u}{\partial r^{2}}\frac{\partial w}{\partial r}+(\frac{\partial w}{\partial r})^{2}\frac{\partial^{2}w}{\partial r^{2}}] \\ +c_{9}h[3\frac{\partial u}{\partial r}\frac{\partial w}{r\partial r}-3\frac{u}{r^{2}}\frac{\partial w}{\partial r}+\frac{1}{r}(\frac{\partial w}{\partial r})^{3}]+\frac{c_{5}h^{3}}{6}\frac{\partial w}{r\partial r}-\frac{c_{7}h^{3}}{6}(\frac{\partial^{4}w}{\partial r^{4}}+\frac{\partial^{3}w}{r\partial r^{3}}) \\ +(\frac{c_{9}h^{3}}{4}+\frac{c_{8}h^{3}}{2})(\frac{\partial^{2}w}{r^{2}\partial r^{2}}-\frac{\partial w}{r^{3}\partial r})+\frac{f_{1}}{\alpha}V_{0}] \end{matrix}$ (B.4)

$S_{w2}(r)=r[\frac{c_{7}h^{3}}{6}\frac{\partial^{3}w}{\partial r^{3}}+\frac{c_{9}h^{3}}{4}(\frac{\partial^{2}w}{r\partial r^{2}}-\frac{\partial w}{r^{2}\partial r})]$ (B.5)

On the basis of the dimensionless parameters defined in Eq. , the dimensionless quantities $\bar{S}_{u}$, $\bar{S}_{u1}$, $\bar{S}_{w}$, $\bar{S}_{w1}$, $\bar{S}_{w2}$ in Eq. are written as

$\begin{matrix} \bar{S}_{u}(\xi)=\xi[\frac{\partial\bar{u}}{\partial\xi}+\frac{\vartheta}{2}(\frac{\partial\bar{w}}{\partial\xi})^{2}+b_{9}\frac{\bar{u}}{\xi}+\frac{b_{2}}{2}(\vartheta\frac{\partial\bar{w}}{\xi\partial\xi}\frac{\partial^{2}\bar{w}}{\partial\xi^{2}}-3\frac{\bar{u}}{\xi^{3}}+3\frac{\partial\bar{u}}{\xi^{2}\partial\xi})+b_{6}[3\frac{\partial\bar{u}}{\xi^{2}\partial\xi}-3\frac{\bar{u}}{\xi^{3}}+\vartheta(\frac{\partial\bar{w}}{\xi\partial\xi})^{2}] \\ -b_{3}(\frac{\partial^{2}\bar{u}}{\xi\partial\xi^{2}}+\frac{\partial^{3}\bar{u}}{\partial\xi^{3}}+\vartheta\frac{\partial\bar{w}}{\xi\partial\xi}\frac{\partial^{2}\bar{w}}{\partial\xi^{2}}+\vartheta(\frac{\partial^{2}\bar{w}}{\partial\xi^{2}})^{2}+\vartheta\frac{\partial\bar{w}}{\partial\xi}\frac{\partial^{3}\bar{w}}{\partial\xi^{3}})] \end{matrix}$ (B.6)

$\bar{S}_{u1}(\xi)=\xi[\frac{\partial^{2}\bar{u}}{\partial\xi^{2}}+\vartheta\frac{\partial\bar{w}}{\partial\xi}\frac{\partial^{2}\bar{w}}{\partial\xi^{2}}+b_{7}(3\frac{\partial\bar{u}}{\xi\partial\xi}-3\frac{\bar{u}}{\xi^{2}}+\frac{\vartheta}{\xi}(\frac{\partial\bar{w}}{\partial\xi})^{2})]$ (B.7)

$\begin{matrix} \bar{S}_{w}(\xi)=\xi[\frac{b_{3}\vartheta}{6}(\frac{\partial^{5}\bar{w}}{\partial\xi^{5}}+2\frac{\partial^{4}\bar{w}}{\xi\partial\xi^{4}}-3\frac{\partial^{3}\bar{w}}{\xi^{2}\partial\xi^{3}}+3\frac{\partial^{2}\bar{w}}{\xi^{3}\partial\xi^{2}}-3\frac{\partial\bar{w}}{\xi^{4}\partial\xi})+\frac{4\vartheta b_{5}}{\xi^{2}}(\frac{\partial\bar{w}}{\partial\xi})^{3} \\ -b_{4}\vartheta(\frac{\partial^{3}\bar{w}}{\partial\xi^{3}}+\frac{\partial^{2}\bar{w}}{\xi\partial\xi^{2}}-\frac{\partial\bar{w}}{\xi^{2}\partial\xi})+2\frac{\partial\bar{u}}{\partial\xi}\frac{\partial\bar{w}}{\partial\xi}+\vartheta(\frac{\partial\bar{w}}{\partial\xi})^{3}+2b_{9}\frac{\bar{u}}{\xi}\frac{\partial\bar{w}}{\partial\xi} \\ +2b_{3}[-\frac{\partial^{3}\bar{u}}{\partial\xi^{3}}\frac{\partial\bar{w}}{\partial\xi}-\frac{\partial^{2}\bar{u}}{\partial\xi^{2}}\frac{\partial\bar{w}}{\xi\partial\xi}-\vartheta\frac{\partial\bar{w}}{\partial\xi}(\frac{\partial^{2}\bar{w}}{\partial\xi^{2}})^{2}-\vartheta(\frac{\partial\bar{w}}{\partial\xi})^{2}\frac{\partial^{2}\bar{w}}{\xi\partial\xi^{2}}-\vartheta(\frac{\partial\bar{w}}{\partial\xi})^{2}\frac{\partial^{3}\bar{w}}{\partial\xi^{3}}] \\ +4b_{6}(\frac{\partial\bar{u}}{\xi^{2}\partial\xi}\frac{\partial\bar{w}}{\partial\xi}-\frac{\bar{u}}{\xi^{3}}\frac{\partial\bar{w}}{\partial\xi})+b_{2}(-\frac{\partial^{2}\bar{u}}{\partial\xi^{2}}\frac{\partial\bar{w}}{\xi\partial\xi}+3\frac{\partial\bar{u}}{\xi^{2}\partial\xi}\frac{\partial\bar{w}}{\partial\xi}-3\frac{\bar{u}}{\xi^{3}}\frac{\partial\bar{w}}{\partial\xi})] \end{matrix}$ (B.8)

$\begin{matrix} \bar{S}_{w1}(\xi)=\xi[b_{4}\frac{\partial^{2}\bar{w}}{\partial\xi^{2}}+b_{8}\frac{\partial\bar{w}}{\xi\partial\xi}+\frac{b_{1}}{6}\frac{\partial\bar{w}}{\xi\partial\xi}+\frac{2b_{3}}{\vartheta}[\frac{\partial^{2}\bar{u}}{\partial\xi^{2}}\frac{\partial\bar{w}}{\partial\xi}+\vartheta(\frac{\partial\bar{w}}{\partial\xi})^{2}\frac{\partial^{2}\bar{w}}{\partial\xi^{2}}]-\frac{b_{3}}{6}(\frac{\partial^{4}\bar{w}}{\partial\xi^{4}}+\frac{\partial^{3}\bar{w}}{\xi\partial\xi^{3}}) \\ +\frac{b_{2}}{\vartheta}[3\frac{\partial\bar{u}}{\partial\xi}\frac{\partial\bar{w}}{\xi\partial\xi}-3\frac{\bar{u}}{\xi^{2}}\frac{\partial\bar{w}}{\partial\xi}+\frac{\vartheta}{\xi}(\frac{\partial\bar{w}}{\partial\xi})^{3}]+\frac{1}{4}(b_{2}+2b_{6})(\frac{\partial^{2}\bar{w}}{\xi^{2}\partial\xi^{2}}-\frac{\partial\bar{w}}{\xi^{3}\partial\xi})+\bar{V}_{0}] \end{matrix}$ (B.9)

$\bar{S}_{w2}(\xi)=\xi[\frac{1}{3}\frac{\partial^{3}\bar{w}}{\partial\xi^{3}}+b_{7}(\frac{\partial^{2}\bar{w}}{\xi\partial\xi^{2}}-\frac{\partial\bar{w}}{\xi^{2}\partial\xi})]$ (B.10)

According to the DQM, the discrete dimensionless quantities $\bar{S}_{u}$, $\bar{S}_{u1}$, $\bar{S}_{w}$, $\bar{S}_{w1}$, $\bar{S}_{w2}$ in Eqs. (B.6)- (B.10) are obtained, respectively, as

$\begin{matrix} \bar{S}_{u}(\xi_{i})=\xi_{i}[\sum_{j=1}^{N} A_{ij}^{(1)}\bar{u}_{j}+\frac{b_{2}}{2}(\frac{\vartheta}{\xi_{i}}\sum_{j=1}^{N} A_{ij}^{(1)}\bar{w}_{j}\sum_{j=1}^{N} A_{ij}^{(2)}\bar{w}_{j}-3\frac{\bar{u}_{i}}{\xi_{i}^{3}}+\frac{3}{\xi_{i}^{2}}\sum_{j=1}^{N} A_{ij}^{(1)}\bar{u}_{j})+b_{1}\frac{\bar{u}_{i}}{\xi_{i}} \\ +\frac{\vartheta}{2}\sum_{j=1}^{N} A_{ij}^{(1)}\bar{w}_{j}\sum_{j=1}^{N} A_{ij}^{(1)}\bar{w}_{j}+b_{6}[\frac{3}{\xi_{i}^{2}}\sum_{j=1}^{N} A_{ij}^{(1)}\bar{u}_{j}-3\frac{\bar{u}_{i}}{\xi_{i}^{3}}+\frac{\vartheta}{\xi_{i}^{2}}\sum_{j=1}^{N} A_{ij}^{(1)}\bar{w}_{j}\sum_{j=1}^{N} A_{ij}^{(1)}\bar{w}_{j}]-b_{3}(\frac{1}{\xi_{i}}\sum_{j=1}^{N} A_{ij}^{(2)}\bar{u}_{j} \\ +\sum_{j=1}^{N} A_{ij}^{(3)}\bar{u}_{j}+\frac{\vartheta}{\xi_{i}}\sum_{j=1}^{N} A_{ij}^{(1)}\bar{w}_{j}\sum_{j=1}^{N} A_{ij}^{(2)}\bar{w}_{j}+\vartheta\sum_{j=1}^{N} A_{ij}^{(2)}\bar{w}_{j}\sum_{j=1}^{N} A_{ij}^{(2)}\bar{w}_{j}+\vartheta\sum_{j=1}^{N} A_{ij}^{(1)}\bar{w}_{j}\sum_{j=1}^{N} A_{ij}^{(3)}\bar{w}_{j})] \end{matrix}$ (B.11)

$\bar{S}_{u1}(\xi_{i})=\xi_{i}[\sum_{j=1}^{N} A_{ij}^{(2)}\bar{u}_{j}+\vartheta\sum_{j=1}^{N} A_{ij}^{(1)}\bar{w}_{j}\sum_{j=1}^{N} A_{ij}^{(2)}\bar{w}_{j}+b_{7}(\frac{3}{\xi_{i}}\sum_{j=1}^{N} A_{ij}^{(1)}\bar{u}_{j}-3\frac{\bar{u}_{i}}{\xi_{i}^{2}}+\frac{\vartheta}{\xi_{i}}\sum_{j=1}^{N} A_{ij}^{(1)}\bar{w}_{j}\sum_{j=1}^{N} A_{ij}^{(1)}\bar{w}_{j})]$ (B.12)

$\begin{matrix} \bar{S}_{w}(\xi_{i})=\xi_{i}[\frac{b_{3}\vartheta}{6}(\sum_{j=1}^{N} A_{ij}^{(5)}\bar{w}_{j}+\frac{2}{\xi_{i}}\sum_{j=1}^{N} A_{ij}^{(4)}\bar{w}_{j}-\frac{3}{\xi_{i}^{2}}\sum_{j=1}^{N} A_{ij}^{(3)}\bar{w}_{j}+\frac{3}{\xi_{i}^{3}}\sum_{j=1}^{N} A_{ij}^{(2)}\bar{w}_{j}-\frac{3}{\xi_{i}^{4}}\sum_{j=1}^{N} A_{ij}^{(1)}\bar{w}_{j}) \\ +(\frac{4b_{5}\vartheta}{\xi_{i}^{2}}+\vartheta)\sum_{j=1}^{N} A_{ij}^{(1)}\bar{w}_{j}\sum_{j=1}^{N} A_{ij}^{(1)}\bar{w}_{j}\sum_{j=1}^{N} A_{ij}^{(1)}\bar{w}_{j}-b_{4}\vartheta(\sum_{j=1}^{N} A_{ij}^{(3)}\bar{w}_{j}+\frac{1}{\xi_{i}}\sum_{j=1}^{N} A_{ij}^{(2)}\bar{w}_{j}-\frac{1}{\xi_{i}^{2}}\sum_{j=1}^{N} A_{ij}^{(1)}\bar{w}_{j}) \\ +2\sum_{j=1}^{N} A_{ij}^{(1)}\bar{u}_{j}\sum_{j=1}^{N} A_{ij}^{(1)}\bar{w}_{j}+2b_{9}\frac{\bar{u}_{i}}{\xi_{i}}\sum_{j=1}^{N} A_{ij}^{(1)}\bar{w}_{j}+2b_{3}\sum_{j=1}^{N} A_{ij}^{(1)}\bar{w}_{j}[-\sum_{j=1}^{N} A_{ij}^{(3)}\bar{u}_{j}-\frac{1}{\xi_{i}}\sum_{j=1}^{N} A_{ij}^{(2)}\bar{u}_{j} \\ -\vartheta\sum_{j=1}^{N} A_{ij}^{(2)}\bar{w}_{j}\sum_{j=1}^{N} A_{ij}^{(2)}\bar{w}_{j}-\frac{\vartheta}{\xi_{i}}\sum_{j=1}^{N} A_{ij}^{(1)}\bar{w}_{j}\sum_{j=1}^{N} A_{ij}^{(2)}\bar{w}_{j}-\vartheta\sum_{j=1}^{N} A_{ij}^{(1)}\bar{w}_{j}\sum_{j=1}^{N} A_{ij}^{(3)}\bar{w}_{j}] \\ +4b_{6}\sum_{j=1}^{N} A_{ij}^{(1)}\bar{w}_{j}(\frac{1}{\xi_{i}^{2}}\sum_{j=1}^{N} A_{ij}^{(1)}\bar{u}_{j}-\frac{\bar{u}_{i}}{\xi_{i}^{3}})+b_{2}\sum_{j=1}^{N} A_{ij}^{(1)}\bar{w}_{j}(-\frac{1}{\xi_{i}}\sum_{j=1}^{N} A_{ij}^{(2)}\bar{u}_{j}+\frac{3}{\xi_{i}^{2}}\sum_{j=1}^{N} A_{ij}^{(1)}\bar{u}_{j}-3\frac{\bar{u}_{i}}{\xi_{i}^{3}})] \end{matrix}$ (B.13)

$\begin{matrix} \bar{S}_{w1}(\xi_{i})=\xi_{i}[b_{4}\sum_{j=1}^{N} A_{ij}^{(2)}\bar{w}_{j}+(\frac{b_{8}}{\xi_{i}}+\frac{b_{1}}{6\xi_{i}})\sum_{j=1}^{N} A_{ij}^{(1)}\bar{w}_{j}+\frac{2b_{3}}{\vartheta}\sum_{j=1}^{N} A_{ij}^{(1)}\bar{w}_{j}[\sum_{j=1}^{N} A_{ij}^{(2)}\bar{u}_{j}+\vartheta\sum_{j=1}^{N} A_{ij}^{(1)}\bar{w}_{j}\sum_{j=1}^{N} A_{ij}^{(2)}\bar{w}_{j}] \\ +\frac{b_{2}}{\vartheta}\sum_{j=1}^{N} A_{ij}^{(1)}\bar{w}_{j}[\frac{3}{\xi_{i}}\sum_{j=1}^{N} A_{ij}^{(1)}\bar{u}_{j}-3\frac{\bar{u}_{i}}{\xi_{i}^{2}}+\frac{\vartheta}{\xi_{i}}\sum_{j=1}^{N} A_{ij}^{(1)}\bar{w}_{j}\sum_{j=1}^{N} A_{ij}^{(1)}\bar{w}_{j}]-\frac{b_{3}}{6}(\sum_{j=1}^{N} A_{ij}^{(4)}\bar{w}_{j}+\frac{1}{\xi_{i}}\sum_{j=1}^{N} A_{ij}^{(3)}\bar{w}_{j}) \\ +\frac{1}{4}(b_{2}+2b_{6})(\frac{1}{\xi_{i}^{2}}\sum_{j=1}^{N} A_{ij}^{(2)}\bar{w}_{j}-\frac{1}{\xi_{i}^{3}}\sum_{j=1}^{N} A_{ij}^{(1)}\bar{w}_{j})+\bar{V}_{0}] \end{matrix}$ (B.14)

$\bar{S}_{w2}(\xi_{i})=\xi_{i}[\frac{1}{3}\sum_{j=1}^{N} A_{ij}^{(3)}\bar{w}_{j}+b_{7}(\frac{1}{\xi_{i}}\sum_{j=1}^{N} A_{ij}^{(2)}\bar{w}_{j}-\frac{1}{\xi_{i}^{2}}\sum_{j=1}^{N} A_{ij}^{(1)}\bar{w}_{j})]$ (B.15)
